# Vegetation Response and Landscape Dynamics of Indian Summer Monsoon Variations during Holocene: An Eco-Geomorphological Appraisal of Tropical Evergreen Forest Subfossil Logs

**DOI:** 10.1371/journal.pone.0093596

**Published:** 2014-04-11

**Authors:** Navnith K. P. Kumaran, Damodaran Padmalal, Madhavan K. Nair, Ruta B. Limaye, Jaswant S. Guleria, Rashmi Srivastava, Anumeha Shukla

**Affiliations:** 1 Palynology and Palaeoclimate Laboratory, Agharkar Research Institute, Pune, India; 2 Centre for Earth Science Studies, Thiruvananthapuram, Kerala, India; 3 Birbal Sahni Institute of Palaeobotany, Lucknow, India; University of Oxford, United Kingdom

## Abstract

The high rainfall and low sea level during Early Holocene had a significant impact on the development and sustenance of dense forest and swamp-marsh cover along the southwest coast of India. This heavy rainfall flooded the coastal plains, forest flourishing in the abandoned river channels and other low-lying areas in midland.The coastline and other areas in lowland of southwestern India supply sufficient evidence of tree trunks of wet evergreen forests getting buried during the Holocene period under varying thickness of clay, silty-clay and even in sand sequences. This preserved subfossil log assemblage forms an excellent proxy for eco-geomorphological and palaeoclimate appraisal reported hitherto from Indian subcontinent, and complements the available palynological data. The bulk of the subfossil logs and partially carbonized wood remains have yielded age prior to the Holocene transgression of 6.5 k yrs BP, suggesting therein that flooding due to heavy rainfall drowned the forest cover, even extending to parts of the present shelf. These preserved logs represent a unique palaeoenvironmental database as they contain observable cellular structure. Some of them can even be compared to modern analogues. As these woods belong to the Late Pleistocene and Holocene, they form a valuable source of climate data that alleviates the lack of contemporaneous meteorological records. These palaeoforests along with pollen proxies depict the warmer environment in this region, which is consistent with a Mid Holocene Thermal Maximum often referred to as Holocene Climate Optimum. Thus, the subfossil logs of tropical evergreen forests constitute new indices of Asian palaeomonsoon, while their occurrence and preservation are attributed to eco-geomorphology and hydrological regimes associated with the intensified Asian Summer Monsoon, as recorded elsewhere.

## Introduction

The buried fossil woods and sub-fossil logs in the wetlands of coastal plains and river banks are the compact plant remains that form an important source of information on environmental changes of the geological and recent past. This compactness entails that wood resists decay and survives on land or in water for a considerably long period before fossilization. More than a century has elapsed since the occurrence of sub-fossil woods in shallow subsurface sediments of recent origin in several parts of India was discovered. One of the oldest descriptions of such occurrences was by Oldham in the early 1900s, when he published the area of recent subsidence. In several parts of India, particularly in coastal areas, the local people are familiar with the occurrence of this buried wood as it is often dug out and used as a source of fuel and timber for making furniture. However, such wood, buried in the shallow subsurface strata, has not attracted adequate attention of geoscientists or dendrochronologists. Our investigations since 1998 in the coastal plains and adjacent hinterland of Kerala State, situated in the southwestern part of India, points out several areas where logs of such fossils are found under thin overburden of Quaternary deposits [1]

Unlike the woods of older geological periods, the Quaternary woods are seldom preserved as petrifactions or permineralized forms. Here wood is fossilized as a charcoal-like material, called ‘fusains’ in the lignites and peat or remains undecayed as a sub-fossil, especially in the Holocene sediments. Considering their ubiquity in the sedimentary sequence, the fossil wood represents a unique palaeoenvironmental database. Such fossil logs do reveal detailed cellular structure and may be compared with their modern analogues. In fact, these fossils form a valuable source of climate data to alleviate a lack of contemporaneous meteorological records, provided they possess annual growth rings. As ‘growth ring formation’ is primarily related to climatic conditions, a specimen of this wood can serve as a potential sample for assessing the pattern of climatic changes in the immediate past few thousand years. This technique has already gained importance in palaeoclimatic study [2]. While studying the Quaternary stratigraphic sequence and geological events of the South Kerala Sedimentary Basin, the authors came across large quantity of tree trunks, embedded in the carbonaceous and silty clays at different stratigraphic levels at several locations along the Kollam-Kodungallur stretch. Therefore, their importance as potential archives of Late Quaternary environment is being addressed here. The natural affinity of the carbonized woods and subfossil logs with their nearest-living-relative forms (NRL), retrieved from the wetlands and associated landforms of southwestern Kerala, has been addressed while appraising the vegetation and climate dynamics of the Holocene period.

## Regional Setting

The area of investigation is restricted to the Southwestern part of India, and it covers the coastal lands <8 m amsl [3] and adjoining parts of the midlands 8–75 m amsl [4] of southern Kerala. The eastern boundary of Kerala State is defined by the Western Ghats mountain range, also called ‘Sahyadri’, and to its west lies the Arabian Sea. This narrow strip of land has a 560 km long coastline. It has an area of 38,863 sq. km and extends between north latitudes 8°17′30″and 12°27′40″ and east longitudes 74°51′57″ and 77°24′47″. The land between the Sahyadri escarpment and the present coast is very narrow. Numerous small rivers originating in the plateau run for a short distance aided by the steep gradient and debouch into the Arabian Sea. Most of these rivers have developed estuaries of various geometries and orientations.

**Figure 1:**
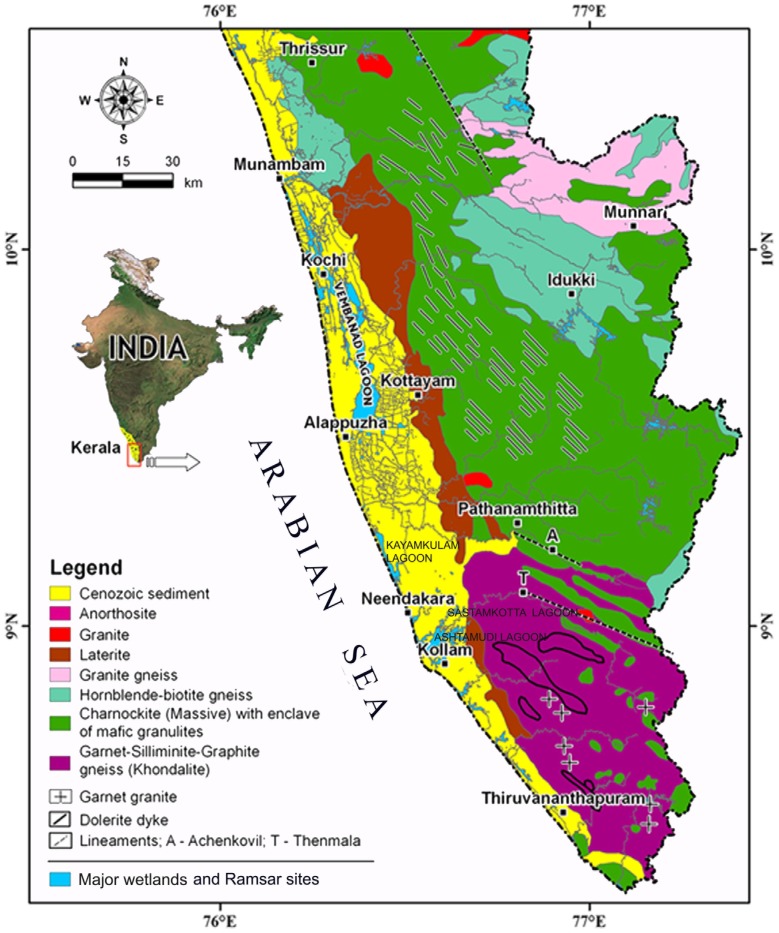
Location of major wetlands in Southwestern India showing geological formations/rock type and lineaments (Modified after [85]).

**Figure 2:**
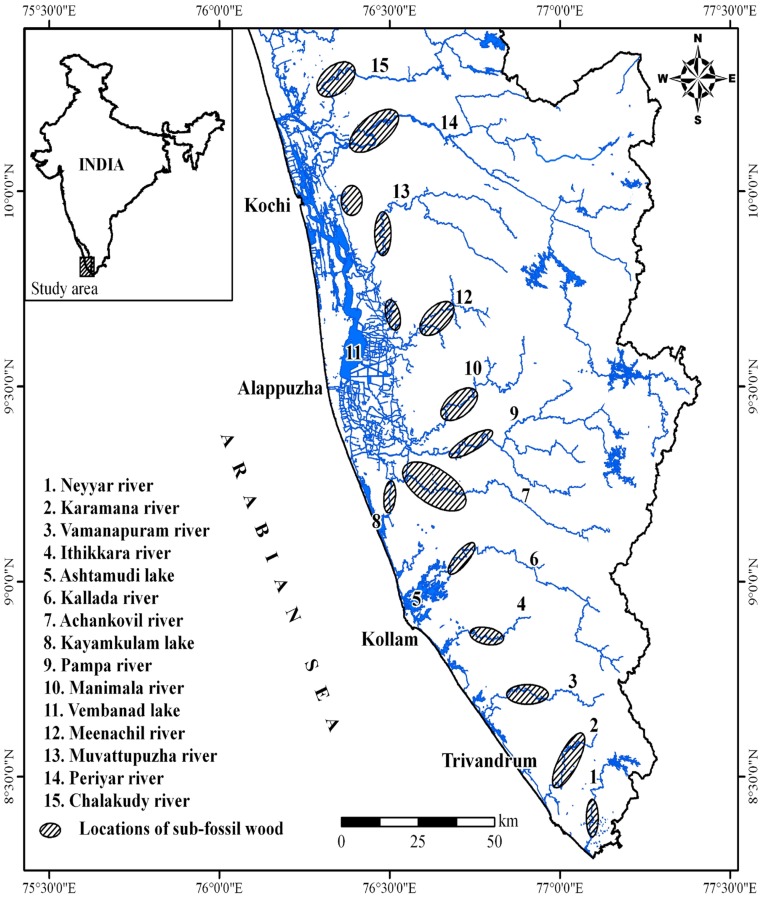
Landward extension of Kerala–Konkan basin showing fossil wood locations associated with wetlands and major river basins of south west India.

Geologically, the study area is composed of the Archean crystallines (Khondalites and Charnockite group of rocks), Neogene sediments (represented by Warkalli and Quilon Formations) and Quaternary deposits (represented by coastal sands, muds and alluvium) (Figure 1). The main part of the coastal land occurs as a curvilinear embayment, which has proved to be a landward extension of the off shore Kerala- Konkan Basin. This landward extension is called the South Kerala Sedimentary Basin (SKSB) [5]. The entire area is drained by 13 small rivers with catchment area <10,000 sq. km [6]. The river valleys in the coastal lands and adjoining midlands are broad with fairly thick alluvial sediments. The lower part of the alluvial sediments is composed of channel sands, while the upper part is made up of silty sediments. The present river channel is narrow as compared to the wide valleys occupied by flood plain sediments. The flood plain sediments of Early Holocene are deposited over riparian vegetation/forests at many stretches. In addition to river valleys, the coastal land close to wetland and areas within wetlands also contain buried woods and sub fossil logs (Figure 2). The area of investigation comprises the coastal plains and the adjoining hinterland between lat. 8°25′ and 10°15′ N. This part of coastal stretch comprises lagoons and estuaries, perennial and seasonal inland wetlands, used for rice cultivation, alluvial fans and fan deltas and the ridge-runnel systems. The coastal plains, located in stretches that are tectonically stable or undergoing uplift, suffer erosion, and consequently no sizeable thickness of sediments can be found in such plains. The Quaternary sediments, therefore, are generally confined to estuaries, abandoned river channels and river terraces.

## Materials and Methods

Approval for field studies was obtained from Directors of Centre for Earth Science Studies, Thiruvananthapuram and Agharkar Research Institute Pune, India. Systematic field studies were carried out to collect data on various landform features in the coastal lands, and regions adjoining the midlands that constituted the study area. The locations of buried wood and sub fossil logs were mapped on 1∶50,000 scale Survey of India Topo base maps. Details of the location of wood samples and their retrieval along with their environment are presented in table 1. Fossil specimens studied, and the micro- preparations in the form of slides are housed in the repository of museum of the Birbal Sahni Institute of Palaeobotany (BSIP), Lucknow, India.

**Table 1:** Location of wood samples along with their environment and details of sample collection.

Sl. No.	Location	Latitude and Longitude	District	Environment	Remarks
1	Elanjikkal	9°16′40″ N–76°34′33″ E	Alappuzha	Wetland	Fossil logs unearthed during clay mining for brick making. The embedding sediment is organic rich clay.
2	Thottappally	9°18′41″ N–76°24′00″ E	Alappuzha	Wetland	Fossil logs retrieved from a wetland near Thottappally junction at 2.0 m depth below ground level. The embedding material is carbonaceous clay.
3	Ayiroor River Section	8°46′10″ N–76°44′01″ E	Thiruvananthapuram	River bank	Wood sample collected from the river bank near Ayiroor which is cut down for constructing bridge. The logs and woods might have been buried under sand and clays.
4	Adichanalloor	9°52′32″ N–76°42′36″ E	Kollam	Wetland	The area (near Adichanalloor junction) from where the wood collected forms a part of the wetland (Kotta *kayal*) which is separated from the Paravur wetland during the progradation of the Ithikkara Bay Head Delta. The woods are buried in organic matter rich black clays.
5	Manimala River–1 (Ambattubhagom)	9°22′38″ N–76°36′05″ E	Pathanamthitta	River channel	Wood samples collected from Manimala river channel near Ambattubhagom. Large tree trunks collected during the process of sand mining.
6	Komallur	9°10′38″ N–76°35′00″ E	Alappuzha	Wetland	Wood samples collected from a fresh sand excavation pit near the north eastern boundary of Komallur wetland. The embedding matrix is organic rich black clay.
7	Manimala River–2 (Karuthavadasserikkara)	9°23′48″ N–76°39′26″ E	Pathanamthitta	River channel	Fossil woods collected from Manimala river channel near Karuthavadasserikkara. Large tree trunk obtained during the process of sand mining.
8	Pathiyur	9°12′07″ N–76°30′37″ E	Alappuzha	Wetland	Fossil logs collected from the Pathiyur wetland. Large tree trunks retrieved during the process of channel construction. The embedding material is organic rich clay.
9	Karamana River (Trikkannapuram)	8°28′30″ N–77°00′00″ E	Thiruvananthapuram	River bank	Fossil logs collected from the river bank near Trikkannapuram. Organic rich layer contains buried wood and logs of various sizes and this horizon is sand-witched between sand layers.
10	Manimala River–3 (Paduthodu)	9°25′14″ N–76°40′12″ E	Pathanamthitta	River channel	Fossil logs collected from the Manimala river channel near Paduthodu during the process of sand mining.

### Specimen details

*Dipterocarpus* sp. cf. *D. indicus* Bedd. (BSIP Museum No. 40078 and BSIP Museum Slide No. 40078–1, 2, 3), *Calophyllum* sp. (BSIP Museum No. 40078 A and BSIP Museum Slide No. 40078 A–1, 2, 3, 4), *Diospyros* sp. cf. *D. bourdilloni* Brandis, BSIP Museum No. 40079 and BSIP Museum Slide No. 40079–1, 2, 3,4,5,6,7), *Careya arborea* Roxb. (BSIP Museum No. 40080 and BSIP Museum Slide No. 40080–1, 2, 3), *Artocarpus* sp. cf. *A. lacucha* Buch-Ham. (BSIP Museum No. 40081 and BSIP Museum Slide No. 40081–1, 2, 3), *Rhizophora* sp. cf. *R. mangle* L.(BSIP Museum No. 40082 and BSIP Museum Slide No. 40082–1, 2, 3) and *Neolamarckia* sp. cf. *N. cadamba* BSIP Museum No. 40083 and BSIP Museum Slide No. 40083–1, 2, 3).

As most of the Quaternary sediments are associated with the wetlands, retrieval of sediment samples and fossil logs was a difficult task. However, trunks, branches and roots of trees buried under 1–5 m of sediments can be retrieved. In addition to this, such vegetal remains are also encountered buried under sand and clay in river beds and flood plains, particularly along river courses in coastal plains and lower elevation of lowlands. Although, the occurrence of carbonized wood and subfossil logs has been observed virtually all along the entire coastal stretch of Kerala, fifteen locations are associated with lake and river basins and palaeoestuaries (Figure 2; Table 1–2). The tree trunks are often found buried under 1–3 m clay sequence in vast areas, all along the coast from Karunagapally to Ernakulam. Along the banks of Kayamkulam and Vembanad lagoons, including Mundakan, Kari, Kuttanad and Kol lands, subfossil logs are encountered while digging canals for local navigation and irrigation. Most of the paddy fields, lakes, swamps and marshes, in the midland region contiguous to SKSB and elsewhere are reported to contain trunks of trees. Some of these wetlands have 6–10 m sediment fills. The clay in some of them is mined for manufacturing of tile-brick. These quarries too expose tree trunks at several levels. Many of the river terraces, particularly those in the terrain, which have undergone uplift in Quaternary times, are also reported to contain carbonized wood. Due to the erosion of the riverbank this wood floats down the river, and is collected to serve as fuel.

**Table 2:** Lithological bearing of the subfossil wood samples along with other relevant details including radiocarbon dates.

Sl. No.	Location	Basin/Environment	Sub - environment	Lithological characteristics	
				Depth (m)	Description **(bgl - Below Ground Level; yrs BP Years Before Present)**
1	Trikkannapuram (KRM-2) 8°28′30″ N–77°0′ E (Thiruvananthapuram district)	Karamana river basin	River bank deposit	0–5.0	Brown to yellowish brown mud dominated sediments
				5.0–9.5	Grey to greyish black clayey sand with decayed vegetative matter and subfossil wood. A wood sample at 9.5 m bgl is ^14^C dated 6970±80 yrs BP.
2	Anikkampi junction (KRM-3) 8°31′30″ N–77°0′20″ E (Thiruvananthapuram district)	Karamana river basin	Over bank deposit (Flood plain)	0–4.0	Yellowish brown sandy mud
				4.0–9.0	Yellowish brown sand dominated sediments with subfossil wood. A wood sample at 8.0 m bgl is ^14^C dated 6140±80 yrs BP.
3	Changa* 8°34′44″ N–77°3′56″ E (Thiruvananthapuram district)	Karamana river basin	Over bank deposit (Flood plain)	0–1.2	Yellowish brown clayey sand
				1.2–3.0	Grey to off white silty sand with decayed wood. A wood sample at 2.5 m bgl is ^14^C dated 3300±90 yrs BP.
4	Aruvikkara (KRM-4) 8°34′27″ N–77°2′35″ E (Thiruvananthapuram district)	Karamana river basin	River bank deposit	0–1.0	Yellowish brown sandy clay
				1.0–4.0	Greyish black, clayey sand with subfossil wood. A sample at 4.0 m bgl is ^14^C dated 1240±10 yrs BP.
5	Perunthrakadavu (VPM-2) 8°43′6″ N–76°53′40″ E (Thiruvananthapuram district)	Vamanapuram river basin	River bank deposit	0–2.5	Brown to yellowish brown mud dominated sediments
				2.5–8.0	Greyish black clayey sand with subfossil wood. A wood sample at 7.0 m bgl is ^14^C dated 10540±110 yrs BP
6	Vamanapuram (VPM-1) 8°43′8″ N–76°54′20″E (Thiruvananthapuram district)	Vamanapuram river basin	Over bank deposit (Flood plain)	0–4.0	Yellowish brown mud dominated sediments
				4.0–7.5	Greyish black sandy clay with decayed vegetative matter and subfossil wood. A wood sample at 4.0 m bgl is ^14^C dated 2910±170 yrs BP.
7	Pangod 9°03′ N–76°42′ E (Kollam district)	Kallada river basin	Over bank deposit (Flood plain)	0–3.0	Yellowish brown mud dominated sediments
				3.0–6.0	Greyish black, organic matter rich silt and sand dominated sediment. Two wood samples at 3.0 and 5.0 m bgl are ^14^C dated 5260±120 yrs BP and 7490±90 yrs BP respectively.
8	Pandalam 9°13′18″ N–76°41′40″ E (Pathanamthitta district)	Achankovil river basin	River bank deposit	0–4.0	Yellowish brown mud dominated sediments
				4.0–6.0	Greyish black clayey sand dominated sediments with subfossil wood. A sample at 6.0 m bgl is ^14^C dated 5560±90 yrs BP.
9	Ramapuram 9°13′30″ N–76°28′55″ E (Alappuzha district)	Coastal land	Palaeo-beach	0–8.0	Medium to fine grained sand with vegetative matter at the bottom. A wood sample at 3.0 m bgl is ^14^C dated 2460±120 yrs BP.
10	Elanjikkal 9°16′40″ N–76°34′33″ E (Alappuzha district)	Coastal land	Wetland	0–3.0	Clayey sand with wood. A wood sample at 2.0 m bgl is ^14^C dated 1840±70 yrs BP.
11	Karippuzha 9°12′46″ N–76°30′21″ E (Alappuzha district)	Coastal land	Wetland	0–3.5	Light grey, coarse to medium grained sand with little clay
				3.5–4.0	Greyish black organic matter rich sandy clay with subfossil wood. A wood sample is ^14^C dated 7140±90 yrs BP.
12	Vettiyar 9°13′5″ N–76°36′15″ E (Alappuzha district)	Coastal land	Wetland	0–3.0	Grey clayey sand with wood. A sample at 2.0 m bgl is ^14^C dated 13880±200 yrs BP.

* Source [86].

The subfossil/non-carbonized/carbonized woods were thin-sectioned using standard Reichert sliding microtome, and the sections made in transverse, tangential longitudinal and radial longitudinal planes were studied under a high power binocular microscope. Systematic affinities of the woods were initially determined by consulting reference literature [7]–[10] and searching the computerized wood database [11]. Subsequent comparisons were made with extant wood samples housed in Xylaria of Forest Research Institute, Dehra Dun and BSIP. Wood descriptions and measurements were taken in accordance with IAWA recommendations [12]. All the figured specimens are deposited in the museum of Birbal Sahni Institute of Palaeobotany, Lucknow, India. Radiocarbon dates of the woods were obtained from Birbal Sahni Institute of Palaeobotany, Lucknow, India. Calibrated age is based on 5570±30 yrs using state of art calib611 version under 2 sigma confidence levels. Though 25 species of woods were identified in the total assemblage, only the diagnostic anatomical details and ecological and climate potential of 7 species are dealt in the main text, and other relevant information and supporting illustrations are referred to in the supplementary data and presented in table 3.

**Table 3:** List of identified sub fossil logs and partially carbonized woods from the wetlands of Kerala.

Sr.no.	Location/Local name/Native name	Identified Genus/species	Family	Wood Characteristics	Modern analogue/Distribution	Ecology/Climate	Remarks with references
1	Thottappally; Anjeli/Aini	*Artocarpus hirsutus* Lam.	Moraceae	Diff use- porous; Growth rings indistinct	Wild Jack or Jungle Jack tropical evergreen tree species, native to India (Karnataka, Kerala, Maharashtra and Tamil Nadu) and endemic to the Western Ghats and found in evergreen forests.	Canopy trees in disturbed evergreen for-ests up to 900 m; Prefers moist, deciduous to partially evergreen woodlands; grows in places with an annual rainfall of >1500 mm	Timber used extensively in construction of ceilings, door frames and furniture. The famous snake boats of Kerala are often hewn out of this wood [33], [49], [87].
2	Thottappally; Plavu	*Artocarpus heterophyllous* Lamk.	Moraceae	Diffuse-porous; Growth rings indistinct	Native to parts of South and Southeast Asia, and believed to have originated in the southwestern rain forests of India; in present day, Kerala, coastal Karnataka and Maharashtra. Jackfruit also found in East Africa, e.g., in Uganda, Tanzania and Mauritius, as well throughout Brazil and Caribbean nations such as Jamaica.	Cultivated at low elevations throughout India, Burma, Ceylon, southern China, Malaya, East Indies, Queensland, Mauritius, Kenya, Uganda and former Zanzibar; it is rare other Pacific islands, as it is in most of tropical America and the West Indies.	Widely used in the manufacture of furniture, doors and windows, in roof construction. The heartwood is used by Buddhist forest monastics in Southeast Asia as a dye, giving the robes of the monks in those traditions their distinctive light-brown color.
3	Thottappally; Barta (Monkey Jack)	*Artocarpus* cf. *A*. *lacucha* Buch.-Ham.	Moraceae	Diffuse-porous; Growth rings indistinct	Tropical evergreen tree; distributed throughout the Indian Subcontinent and Southeast Asia	Moist evergreen forest, mixed deciduous forest, dry deciduous forest	Valued for its wood.
4	Elanjikkal; Sample no. 1; (**Ebony**)	*Diospyros* sp. cf. *D. bourdilloni*	Ebenaceae	The heartwood is dark, sometimes with black stripes or streaking and often with a greenish cast in sharp contrast to the white to straw colored sapwood.	Native to the tropics; former Maui Nui in Hawaii, Caspian Hyrcanian mixed forests, Kathiarbar-Gir dry deciduous forests, Louisiade Archipelago rain forests, Madagascar lowland forests, Narmada Valley dry deciduous forests, New Guinea mangroves or South Western Ghats, montane rain forests.	lowland dry forests	Timber divided into two groups: the pure black ebony (notably from *D. ebenum*, but also several other species), and the striped ebony or Calamander wood (from *D. celebica*, *D. mun* and others). Most species in the genus produce little to none of this black ebony-type wood; their hard timber (e.g. of American Persimmon, *D. virginiana*) may still be used on a more limited basis.
5	Karamana River (Trikkannapuram), Pathiyur; Aima, Karekku (tamil)Alam, Paer, Peelam, Pela (Malayalam)	*Careya arborea* Roxb.	Lecythediaceae	Medium-weight to heavy hard wood; Heartwood pale red to dark red-brown in older trees, sapwood wide, pale reddish-white; grain straight; texture medium and even. Growth rings distinct	Common in semi-open forests and disturbed areas; deciduous tree that grows up to 15 m–45 ft high; grows throughout India in forests and grasslands. *C. arborea* is the only Malaysian species, which is found almost throughout the range of the genus, but in Peninsular Malaysia it is rare and only occurs in the northwest. It occurs on well-drained, sandy or even rocky soils.	Found scattered but is locally common in primary or secondary, evergreen or deciduous, slightly seasonal forest, sometimes in more open country and along forest edges, and is absent from per humid rain forest.	The wood of *C. arborea* is used, mainly in India and Burma (Myanmar), for general construction (house posts, planking), furniture and cabinet work, carts, mouldings, turnery, piling and agricultural implements.
6	Adichanalloor (Red Mangrove)	*Rhizophora* sp. *R. mangle*	Rhizophoraceae	Woods have high density	Native to American west and east coasts and African west coast. One species,*Rhizophora mangle*, was introduced to the central Pacific, including Hawaii and the Society Islands.	Distributed in estuarine ecosystems throughout the tropics. Inhabits the intertidal wetland zone, 0–6.0 m elevation between mean sea level and highest tides, with variable rainfall.	Well adapted to salt water, they thrive where many other plants fail and create their own ecosystems; recognized as valuable timber producers, beneficial to shoreline stabilization and fisheries
7	(Red mangrove; Asiatic mangrove)	*Rhizophora mucronata* Lam.	Rhizophoraceae	Very hard and termite-resistant wood; Growth rings distinct	Is a species of mangrove found on coasts and river banks in the Indo-Pacific region. In India, it grows in association with the mangrove *Avicennia officinalis*, the golden leather fern (*Acrostichum aureum*) and the sea holly(*Acanthus ilicifolius*). *R. mucronata* is native to **Africa** (in southeastern Egypt; eastern Ethiopia; eastern Kenya; Madagascar; Mauritius; Mozambique; the Seychelles; Somalia; eastern Kwa Zulu-Natal in South Africa; southeastern Sudan; and eastern Tanzania); **Asia** (in Burma; Cambodia; India; Indonesia; the Ryukyu Islands of Japan; Malaysia; Pakistan; Papua New Guinea; the Philippines; Sri Lanka;Taiwan; Thailand; and Vietnam) the **South Pacific** (in the Solomon Islands; and Vanuatu) and **Australia** (in northern Northern Territory; and northern Queensland)	This species is found in the intermediate to upstream estuarine zone in the lower to mid-intertidal region, and more to the seaward side. This species tolerates a maximum salinity of 40 ppt and a salinity of optimal growth of 8–33 ppt.	It is used to help prevent coastal erosion and in restoration of mangrove habitats; the timber is used for firewood and in the construction of buildings, as poles and pilings, and in making fish traps [49].
8	Vellaikadambu (Tamil) Kadamb	*Neolamarkiana* sp. *N. cadamba*	Rubiaceae	Lightweight hardwood with poor durability.	An evergreen, tropical tree native to South and Southeast Asia.	Deep, moist, well-drained loamy soils of alluvial origin.	The timber is used for plywood, light construction, pulp and paper, boxes and crates, dug-out canoes, and furniture. Kadamba yields a pulp of satisfactory brightness and performance as a hand sheet. The wood can be easily impregnated with synthetic resins to increase its density and compressive strength.
9	Lagarto caspi	*Calophyllum* sp.	Clusiaceae/Guttiferae	Wood diffuse porous; Growth ring boundaries distinct. Heartwood basically brown to red to white or grey. Sapwood colour distinct from heartwood colour. Density 0.43–0.6–0.8 g/cm^3^.*Calophyllum* heartwood is a light reddish-brown. Texture is intermediate to coarse and the grain is generally interlocked.	Species grow in a wide number of habitats, from ridges in mountain forests to coastal swamps, lowland forest. Widely distributed throughout Southeast Asia on sites they range from coastal and swamp to mountain forests. Geographic distribution: India, Pakistan, Sri Lanka, Burma, Thailand, Laos, Vietnam, Cambodia, and Indo Malaysia.	Species grow in a wide number of habitats, from ridges in mountain forests to coastal swamps, lowland forest and even coral caves.	Flooring, furniture components, light construction, boat-building, cabinetwork. The upland species are heavier and superior in strength and durability to floodplain species; used for making boats, timber and construction [45].
10	Kunthirikkam (Pantham, Thalli) (Black Dammar)	*Canarium strictum*	Burseraceae	Grayish-white with a pinkish cast to the heartwood; Growth rings indistinct	Native to India and Myanmar; in the Western Ghats- South and Central Sahyadris; large evergreen trees; tropical and subtropical trees native to tropical Africa, southern Asia, and Australia, from southern Nigeria east to Madagascar, Mauritius, India, southern China, Indonesia and the Philippines.	Occasionally canopy trees in the evergreen forests up to 1600 m.	Used for making boards for ceiling, flooring and partitions from well seasoned timber. It is also used for packing cases and for cheap utility furniture. The wood has good glue holding capacity and used for plywood tea-boxes [88].
11	Eetty, internationally known as “Indian Rosewood”.	*Dalbergia latifolia* Roxb.	Fabaceae	The sapwood of *D. latifolia* is pale yellowish-white often with a tinge of purple. Heartwood varies in color from light golden brown to shades of light purple with dark streaks, or deep purple with distant black lines. The heartwood darkens with age. Initial parenchyma cells, Growth rings fairly distinct.	The natural range of *Dalbergia latifolia* stretches from the sub-Himalayan tract to the southern tip of India and the island of lava in Indonesia Best growth occurs in the Western Ghat forests of Karnataka, Kerala, and Tamil Nadu. Introduced to Burma, Sri Lanka, Nepal, Nigeria, and Kenya	This species grows on a variety of soil formations including; gneiss, trap, laterite, alluvial, and boulder deposits. It grows best on well-drained, deep, moist soils. *Dalbergia latifolia* is common on deep loams or clays containing lime. It also grows well on black cotton soils. Shallow dry soils and poor drainage stunt tree growth.	Used to manufacture furniture, paneling, and other ornamental products. Medicines and an appetizer are made from tannins in the bark.
11	Kambagam	*Hopea parviflora* Bedd.	Dipterocarpaceae	Growth rings indistinct	Endemic to the Western Ghats-South and Central Sahyadris. Evergreen, semi-deciduous and deciduous moist forest from sea level to about 900 m.	Common emergent to canopy trees in evergreen forests, up to 900 m.	The wood is finely grained, very durable, and used for making boats, bridges, and furniture.
12	Manimala River–2 (Karuthavadasserikkara); Keruing (Gurjan)	*Dipterocarpus* sp cf. *D. indicus*	Dipterocarpaceae	Heartwood varies from pinkish brown to red brown or dark brown, sometimes with a purple tint, darkens with age, often with distinct resinous odour; sapwood grey-brown, well defined.	Indonesia, Malaysia, Philippines, Sabah, Sarawak, Brunei, Pakistan, India, Myanmar, Borneo, Thailand, Sri Lanka and Kampuchea.	Coastal to inland, riverine to swampy and to dry land, undulating to level terrain, ridges, slopes, valley bottoms, soils deeply weathered to shallow, well-drained to poorly drained, and rich to poor in nutrients.	Suitable for plywood and veneer, container flooring, general construction work, railway sleepers, bridges, harbor work, wagons, truck bodies etc.
13	Elanjhi, elengi (Bulletwood)	*Mimusops elengi* L.	Sapotaceae	Diffuse- porous Fairly distinct; Growth rings indistinct	West coast tropical evergreen forest; dominant in sacred groves; India, Hawai, Australia. Along the ravines.	Tropical evergreen forest	Building and bridge construction; boat- building; furniture and cabinets; agricultural implements; musical instruments; tool handles; turnery and carvings.
14	Vengha, Vengai; (Indian Kino Tree)	*Pterocarpus marsupium* Roxb.	Fabaceae	Wood very hard, close-grained, giving a red resin: sapwood small; heartwood yellowish-brown, with darker streaks. The heartwood is full of resin and stains yellow when damp. Growth rings distinct to indistinct	Widely distributed in central, western and southern regions of India; grown in deciduous and evergreen forests of central, western and southern regions of India. Found mostly in the states of Gujarat, Madhya Pradesh, Bihar and Orissa; Central and Southern India, chiefly in deciduous forest, extending north to the hills of Bihar, Banda, to the Kumaon Terai, low country of Ceylon.	Dry zone	The heart wood is used as an astringent and in the treatment of inflammation and diabetes. anti diabetic preparations, marketed in India, containing *Pterocarpus marsupium* among other ingredients [89]
15	Odal, Vellayodal, Odal,	*Sarcostigma grandis/kleinii* Wight & Arn.	Icacinaceae	Growth ring boundaries usually indistinct to completely absent; Diffuse-porous; Solitary vessels, scalariform perforations with many bars and long vessel elements, as well as taxa that possess a so-called derived set of wood features, such as a high frequency of vessel groupings, simple perforations and short vessel elements, are present.	Throughout the dense forests in the Western Ghats.	Evergreen to semi evergreen; moist deciduous	Plant pacifies vitiated vata, arthritis, anorexia, worm infestation, skin diseases, hysteria, epilepsy, ulcers, and headache.
16	Puli	*Tamarindus indica* L.	Fabaceae	Tamarind timber consists of hard, dark red heartwood and softer, yellowish sapwood. Growth rings distinct to indistinct	Indigenous to tropical Africa. particularly in Sudan, where it continues to grow wild; it is also cultivated in Cameroon, Nigeria and Tanzania; in Arabia, it is found growing wild in Oman, especially Dhofar, where it grows on the sea-facing slopes of mountains. It reached South Asia likely through human transportation and cultivation several thousand years prior to the Common Era; It is widely distributed throughout the tropical belt, from Africa to South Asia, Northern Australia, and throughout South East Asia, Taiwan and China.	Grows well in full sun in clay, loam, sandy, and acidic soil types, with a high drought and aerosol salt (wind-borne salt as found in coastal area) resistance.	*Tamarindus* is a monotypic taxon, having only a single species. tamarind is best described as sweet and sour in taste, and is high in acid, sugar, B vitamins and, oddly for a fruit, calcium. Tamarind wood is a bold red color. Due to its density and durability, tamarind heartwood can be used in making furniture and wood flooring.
17	Thekku, (Teak)	*Tectona grandis* L.F.	Verbenaceae/Lamiaceae	Distinct growth rings	*Tectona grandis* is native to India, Indonesia, Malaysia, Myanmar, northern Thailand, and northwestern Laos; naturalized and cultivated in many countries, including those in Africa and the Caribbean. Burma accounts for nearly one third of the world's total teak production.	Found in a variety of habitats and climatic conditions from arid areas with only 500 mm of rain per year to very moist forests with up to 5,000 mm of rain per year.	Extensively used in India to make doors and window frames, furniture, and columns and beams in old type houses.
18	Pillamaruthu	*Terminalia paniculata* Roth.	Combretace-ae	Wood pale brown, smooth; Growth rings indistinct	Peninsular India; in the Western Ghats - throughout. native to southwest India	Along margin or in the openings of evergreen and semi-evergreen forests, up to 1200 m.	Timber very useful for ship building & is used as substitute for teak.
19	Agil, (Australian red cedar)	*Toona ciliate* Roem.	Meliaceae	The timber is red in colour; Growth rings indistinct.	Grows throughout southern Asia from Afghanistan to Papua New Guinea and Australia. Also occurs in Asia and Malaysia.	Altitudinal range from near sea level to 1000 m.	Used extensively for furniture, wood panelling and construction, including shipbuilding
20	Mavu	*Mangifera indica* L.	Anacardiaceae	Diffuse - porous; Fairly distinct	West coast tropical evergreen and West coast semi-evergreen forests	Hot and Humid conditions	Ceiling boards, window frames; general purpose Class I plywood; furniture and cabinets; block boards; match splints and boxes; boat and shipbuilding; bobbins; bentwood articles
21	Valiyacheru; Anacheru	*Holigarna* sp.	Anacardiaceae	Diffuse-porous; Indistinct	West coast tropical evergreen forest	Evergreen with high rainfall	Packing cases and boxes; match splints [45]).
22	Hog plums, Spanish plums, libas	*Spondias* sp.	Anacardiaceae	Wood diffuse porous; growth rings indistinct	Native to the Neo tropics and about 10 are native to tropical Asia.	Near shore, coastal regions	[45].
23	Indian Ash Tree; shinti (Odiar, Gumphini in South India)	*Lannea coromandelica* (Houtt.) Merr.	Anacardiaceae	Wood diffuse porous; growth rings indistinct; heartwood is rich in leucocyanidin.	Well distributed in India; Throughout Bangladesh, also cultivated as live fence and hedge.	Tropical moist deciduous; restricted to high rainfall regions	The bark is considered astringent and stomachic; used as a lotion in impetigenous eruptions, leprous and obstinate ulcers; cures sprains, bruises, skin eruptions, heart diseases, dysentery and mouth sores. Decoction of the bark is used for toothache [45].
24	Maramamaram	*Sonneratia apetala* Buch–Ham.	Sonneratiaceae	Wood diffuse porous; growth rings not seen.	In India both the coast from Bombay to Sunderbans. In Sri Lanka, Koddiyarstem island. And in Myanmar, Moulmein.	Gregarious along the intertidal estuarine regions of mangrove forests, often as a pioneer species on newly formed mudflats; Marine	Used for paper pulp, matches, and as poles [45].
25	Rajbrikh, kani–konna (Amaltas, golden shower tree)	*Cassia fistula* Linn.	Fabaceae	Sapwood greyish-white to light yellowish- brown, heartwood yellowish-red to brick red or reddish-brown Diffuse-porous; Fairly distinct	Native of tropical Asia, widely cultivated and naturalized in the tropics including West Indies and continental tropical America. It is associated with the Mullai region of Sangam landscape.	Ranging from Tropical Thorn to Moist through Subtropical Thorn to Moist Forest Life Zones	Strong, very durable wood, and used to construct “Ahala Kanuwa”, a place at Adams Peak, Sri Lanka building construction; plough- handles; wheels and shafts of carts; turnery; tool handles; charcoal [49].

## Observations and Results

### Mode of occurrence of fossil wood and subfossil logs

The subfossil logs and partially carbonized wood remains were excavated from trenches, and also retrieved from subsurface sediments of landforms in varied environmental settings. They have been found in wetlands adjacent to lagoonal water bodies, as well as those away from coast without any physiographic connection to the present lagoons. In both cases, tree trunks are found buried under clay, usually rich in carbon. The trunks are found with roots and branches still intact, indicating clearly that the trees grew and fell down at the same place, and got buried under sediments. Besides, no geological agent appears to have been capable of transporting such large trunks with diastemising roots and branches. The sediments in which the trunks occur are found to be of the same age or ages younger by 100–200 years. These sediments are rich in microflora, supportive of inland swamps/marshes, or in some cases of mangrove origin with salinity tolerant varieties [13]–[14]. The sediments with salt tolerant microflora, which mostly contain littoral micro fauna possibly washed in by tidal action, also store preserved hard tree trunks.

Tree trunks of the past vegetation are also preserved in wetlands that lie at a distance of 10–20 km away from the coast. These wetlands have no physical contiguity, in the form of embayment, with multiple digits that contain laterites or sand mounds on the sides. The clay in such wetlands is usually 2–5 m thick and rich in carbon. Carbonized wood fragments are rich in this type of environmental setting. Tree trunks with roots and branches are noticed at different locations of these sand mounds. It is apparent then that the laterites and sand mounds were covered by thick forest, yielding abundant organic matter deposited with the clay.

The sediments in the river channels and adjacent paleo-flood plains are underlain by a horizon with uprooted tree trunks. These get exposed when the 4–5 m thick sand in the river channels is mined. The paleo - flood plains also include meandering river channels where sand mining exposes buried tree trunks. This wood undergoes fragmentation on exposure with the flowing river bed, and the resultant fragments are collected by locals to serve as cooking fuel. Preservation of fossil wood and sub fossil logs is directly related to the sediments in which they are found. The best preservation with minimal carbonization and disintegration is found in cases where the wood is covered by pure clay. If the clay is highly sandy/silty, the wood undergoes carbonization at an increased pace. In fact, in most cases, the clay is sandy, resulting in various degrees of carbonization. In rare cases, wood has been found at the interface between a lower sandy layer and an upper clayey horizon. In such cases, the wood appears to have undergone complete carbonization with overburden pressure; the carbonized trunk appears highly fragmented, devoid of any internal structure. Therefore, it appears that preservation of wood is a function of the sediment which encases it. The inference, therefore, is that only a small part of the wood that grew in an area is preserved.

### Field observation and chronology of subfossil logs

**Figure 3:**
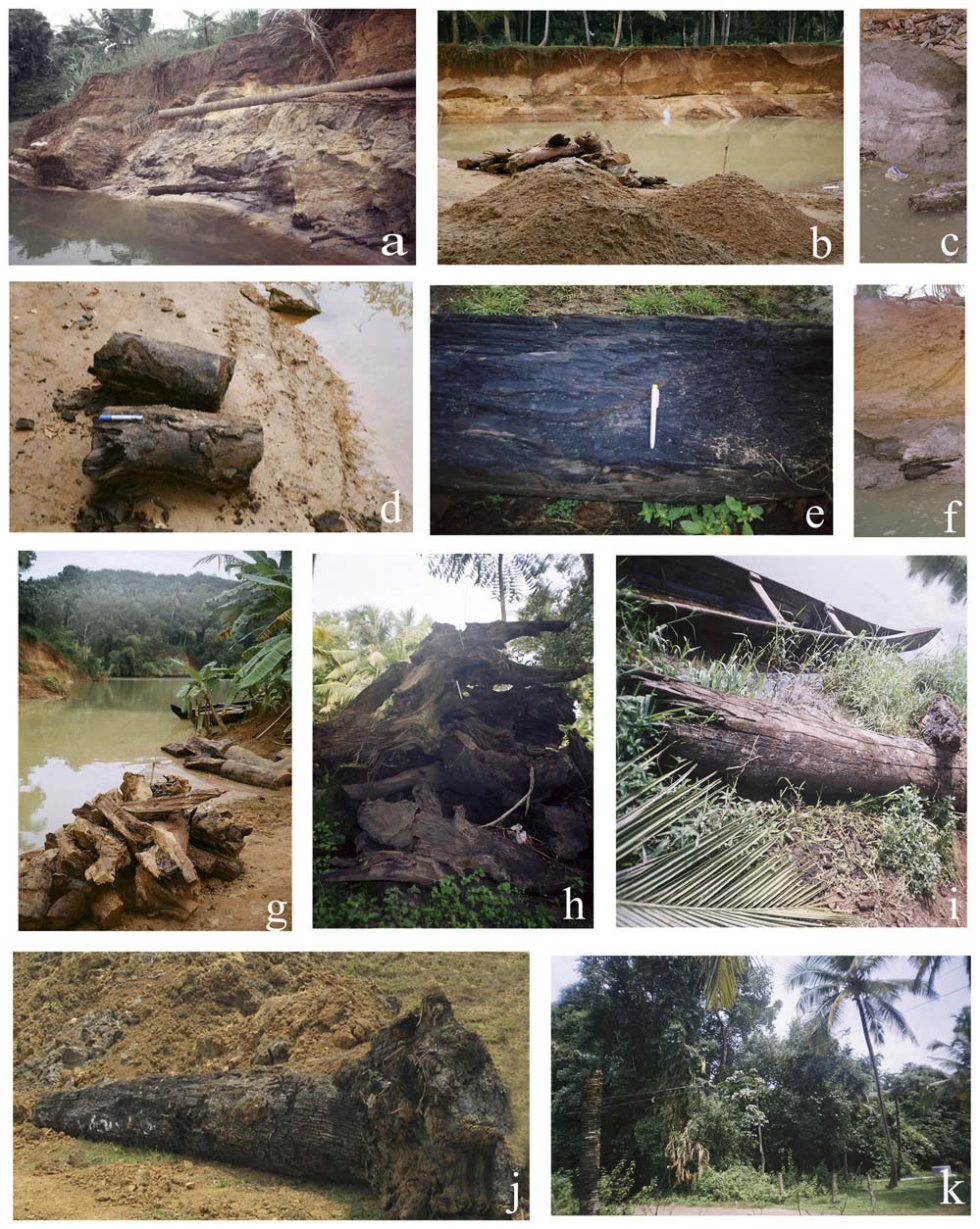
Field view of subfossil logs from wetlands of Kerala. (a) Vamanapuram River Bank, Thiruvananthapuram, ∼3 km west of Parippally. (b), (d) and (g) Karamana River bank, Thiruvananthapuram. (c) and (f) Ayiroor. (e) Partially carbonized wood from Karippuzha, Alappuzha. (h) Heap of carbonized logs in the backyard of a coastal village, Pathiyoor. (i) Vettiyar, Alappuzha. (j) Coastal lowlands of Periyar River. (k) Sacred grove of Keerikkad village, Alappuzha.

Considering the radiocarbon ages, the collected fossil wood and sub fossil log samples can be grouped into three categories. The oldest category belongs to Late Pleistocene- Early Holocene (13.0–10.0 k yrs BP) and the fossils are from Vettiyar and Vamanapuram River bank, west of NH 47. Their occurrence can be attributed to the intensified monsoon of *ca* 13 and 10 k yrs BP. The pattern of flooding and uprooting of roots along the river banks and entombment of the tree trunks occurred soon thereafter. The second category includes the wood remains of ∼8.0 to 5.3 k yrs BP retrieved from wetlands adjacent to lagoonal water bodies and abandoned meander loops. The tree trunks in this setting are embedded in clayey sand/sandy clay and exhibit evidence of abrupt flooding that led to uprooting of trees, and burial under sediments of approximately the same age. The samples from Pangod quarry, Karippuzha ‘punja’, Panthalam (Achankovil River bank) and Karamana River bank belong to this category. The fallen trees were buried by excessive sediment deposited by the rivers. Samples from Vamanapuram River bank to the east of NH 47 and Killiar bank represent the present day vegetation and climate (Figure 3).

### Systematic description of identified woods

Seven taxa were anatomically recognized among the 25 species of wood assemblage (Table 2). Anatomical literature and illustrations of earlier published works were used for identification, and sub fossil logs were assigned to their NRL [7]–[10], [15]–[31]. All the taxa are briefly described to show the basis of identification, affinity and ecological and climate potential. The antiquity of the described species and their present distribution has also been dealt with. Anatomical description of the identified woods and supporting illustrations are provided in the supplementary files.

#### Artocarpus sp. cf. A. lacucha

Buch-Ham., Moraceae, Figure A,1–5 in File S1; Figured Specimen–BSIP Museum No. 40081.

Carbonized wood pieces were collected from Thottapally wetland (9°18′41″ N–76°24′00″ E) in Alappuzha. The wood samples are well preserved and show all the anatomical details.

The important characters of the present fossil are: presence of medium to large vessels, which usually occur alone or in radial multiples of 2–4 with abundant tyloses; large inter-vessel pits, vasicentric to aliform-confluent parenchyma, heterocellular broad rays with occasional occurrence of latex tubes and non-septate fibres. These xylotomical features indicate its close affinity with the modern woods of the genus *Artocarpus* Forster and Forster f. of the family Moraceae. In order to find their nearest modern counterpart, thin sections available at Birbal Sahni Institute of Palaeobotany and photographs of a number of *Artocarpus* species were critically examined, viz., *A. chaplasha* Roxb., *A. dadah* Miq., *A. elasticus* Reinw. ex Blume, *A. gomezianus* Wall. ex Trec., *A. heterophyllus* Lamarck., *A. hirsutus* Lamarck., *A. incise* Linn., *A. lacucha* Buch-Ham., *A. lancefolius* Roxb., *A. nitidus* Trec. *A. scortechinii* King, *A. sericarpus* Jarrett, *A. sepicanus* Deils and *A. tomentosulus* Jarrett. The present carbonized wood shows resemblance with *A. chaplasha, A. gomezianus* and *A. lacucha* which are xylotomically inseparable [20]. Amongst them, *A. lacucha* is found in Kerala [25]. In view of its close anatomical similarity and its occurrence in the Kerala, the sub-fossil has been assigned to *A. lacucha*.

The family Moraceae is a small family with 38 genera and 1100 species distributed in tropical warm regions with a few in temperate zones. Of the five tribes of Moraceae, the genus *Artocarpus* Forster J. R. and Forster belongs to the tribe Artocarpeae. About 50 species of this genus are distributed in Indo-Malaysian region [26]. About 7–8 species are found in India, of which *A. heterophyllus* Lamarck, *A. hirsutus* Lamarck and *A. lacucha* Buch-Ham. are distributed in the moist evergreen forests of Western Ghats.

The fossil history of the genus *Artocarpus* dates back to Maastrachtian-Danian in India, and a large number of fossils of different plant parts have been recorded from different Neogene sequences of the world [32]. The genus has continued to occur along the Kerala coast since Miocene i.e., about 15 million years ago [32]–[34].

**Figure 4:**
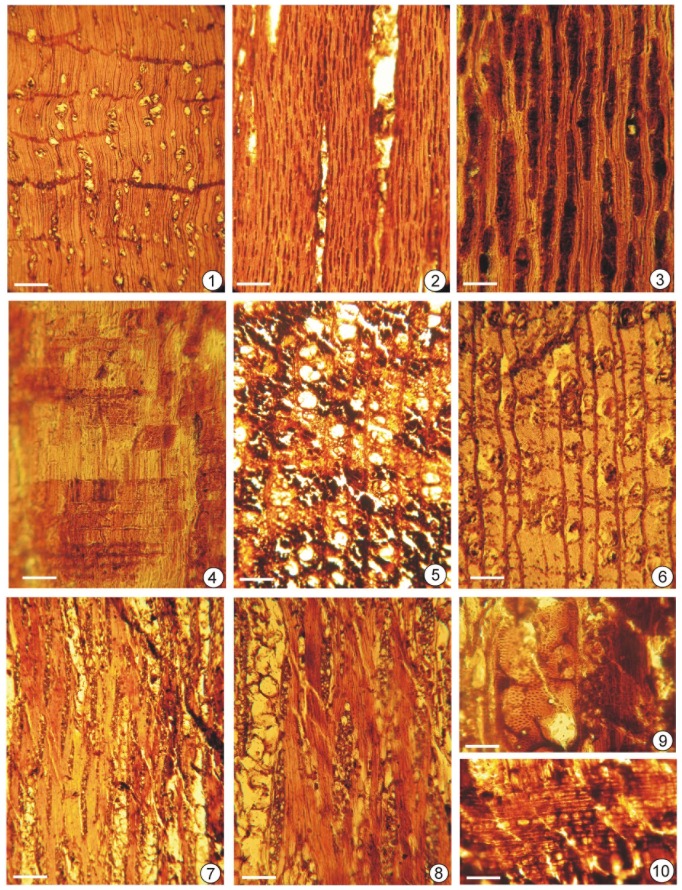
Wood anatomical details of *Calophyllum* sp. (1–4) and *Careya arborea* Roxb. (5–10). 1. Transverse section showing distribution of obliquely arranged tylosed vessels and apotracheal parenchyma bands. Scale bar  = 250 µm; (BSIP Museum Slide No. 40078 A-1). 2. Tangential longitudinal sections showing uniseriate rays and tylosed vessel elements. Scale bar  = 100 µm; (BSIP Museum Slide No. 40078 A-2). 3. Tangential longitudinal sections magnified showing uniseriate rays ands solitary crystal Scale bar  = 50 µm; (BSIP Museum Slide No. 40078 A-3). 4. Radial longitudinal section showing heterocellular rays. (BSIP Museum Slide No. 40078 A-4). 5. Transverse section showing distribution of tylosed vessels and apotracheal parenchyma lines. Scale bar  = 250 µm; BSIP Museum Slide No. 40080-1. 6. Transverse section of another sample showing distribution of tylosed vessels and apotracheal parenchyma lines. Scale bar  = 250 µm; BSIP Museum Slide No. 40080-2. 7. Tangential longitudinal section showing distribution of multiseriate rays and tylosed vessels. Scale bar  = 250 µm; BSIP Museum Slide No. 40080-3. 8. Tangential longitudinal section enlarged of another sample showing a multiseriate rays. Scale bar  = 100 µm; BSIP Museum Slide No. 40080 - 4. 9. Tangential longitudinal section showing bordered, alternate hexagonal intervessel pits. Scale bar  = 50 µm; BSIP Museum Slide No. 40080 -5. 10. Radial longitudinal section showing heterocellular ray cells. Scale bar  = 100 µm. BSIP Museum Slide No. 40080 - 6.

***Calophyllum*** sp., Clusiaceae, Figure 4, 1–4; (see Text S1 in File S1 for detailed description).

The characteristic features of the fossil wood are: solitary vessels with tyloses arranged in oblique radial lines, vasicentric tracheids, apotracheal parenchyma in broken bands and uniseriate rays. These characters collectively indicate affinities of the wood sample with the extant woods of the genus *Calophyllum* Linn. of the family Clusiaceae.

The genus *Calophyllum* Linn. is confined to moist tropical regions of the world mainly in Southeast Asia. About a dozen species occur indigenously in India and it is difficult to distinguish them xylotomically from each other. Hence the sample is assigned to *Calophyllum* sp. Out of a dozen species, *Calophyllum inophyllum* Linn., *C*. *tomentosum* Wight and *C*. *wightianum* Wall. are found on river banks and evergreen forests of Kerala. *C. inophyllum* is found along the coast above high water marks.

***Careya arborea*** Roxb., Lecythidaceae, Figure 4, 5–10 and Figure B, 1–6 in File S1, Figured Specimen–BSIP Museum No. 40080.

The description is based on two pieces of woods retrieved from Karamana River bank (Trikkannapuram - 8°28′30″ N–77°00′00″ E) in Thiruvananthapuram and Pathiyur wetland (9°12′07″ N–76°30′37″ E) in Alappuzha.

The characteristic features of the fossil wood are: vessels mostly in radial multiples, simple perforations, intervessel pits medium-large; abundant parenchyma, both paratracheal and apotracheal; paratracheal vasicentric and apotracheal as diffuse-in-aggregate forming 1–2 seriate broken lines; rays 1- 4(5) seriate, heterocellular and fibres nonseptate. These characters collectively indicate that the fossil logs possess a close resemblance with modern woods of *Careya* Roxb. from the family Lecythidaceae. On surveying the anatomical literature and examining the available modern wood slides, it is found that the samples exhibit strong resemblance with the extant woods of *Careya arborea* Roxb. Pantropical family Lecythidaceae consists of about 24 genera and 285 species distributed across tropical rain forests, especially South America. *Careya* is a small genus (4 species) of trees and shrubs found in the Indo-Malaysian region. *Careya arborea* Roxb., which resembles the wood samples, is a moderate sized tree and widely distributed across India including West Coast (Karnataka, Kerala and Tamil Nadu States) and Myanmar. Hence it is assigned to the same species. The genus *Careya* is well documented from the Neogene exposures of India. The genus has continued to occur on the Kerala coast since Miocene times [35].

***Diospyros*** sp. cf. ***D. bourdilloni*** Brandis, Ebenaceae, Figure C, 1–7 in File S1, Figured Specimen–BSIP Museum No. 40079.

The carbonized wood samples were collected from Elanjikkal wetland (9°16′40″ N–76°34′33″ E) in Alappuzha.

The diagnostic features of the wood are: presence of mostly small to medium sized vessels in radial multiples of 2–5, scanty paratracheal parenchyma, apotracheal forming uniseriate concentric lines at regular intervals; frequent uniseriate rays, rare biseriate due to pairing of cells, composed of procumbent cells in the centre with uniseriate extensions of 1–2 upright cells at both the ends and non-septate, thick walled fibres. These features suggest close affinity of the carbonized wood with the genus *Diospyros* L. of the family Ebenaceae. The study revealed that though xylotomical characters of most species are very similar; yet the carbonized wood under consideration closely resembles *D. bourdilloni* Brandis and *D. varigata* Kurz in all structural details, owing to the frequent occurrence of biseriate rays [10].

The small family Ebenaceae consists of only 2 genera (*Diospyros* L. and *Euclea* Murr.), and 485 species distributed across tropical and sub-tropical regions of the world. Of the 475 species of genus *Diospyros*, 200 occur in Indo-Malaysian region [26]. About 40 species are found in India. *D. bourdilloni* -the nearest modern counterpart is found in the moist tropical evergreen forests of Anamalai and hills of Tirunelveli up to an elevation of 600 m [23], while *D. varigata* is distributed across the north-eastern states (Assam, Meghalaya and Mizoram).The fossil woods of genus *Diospyros* are known to exist from Maastrachtian-Danian onwards in the world, and occur frequently in Neogene exposures in India. The genus has continued to occur in South India (Kerala and Tamil Nadu) since Miocene times [32]–[34].

***Dipterocarpus*** sp. cf. ***D. indicus*** Bedd., Dipterocarpaceae, Figure D, 1–5 in File S1, Figured Specimen–BSIP Museum No. 40078.

The species is based on carbonized wood piece retrieved from river channel deposits of Manimala River - 2 (Karuthavadasserikkara: 9°23′48″ N–76°39′26″ E) Pathanamthitta.

The diagnostic features of the wood are: almost solitary tylosed vessels, vasicentric tracheids, scattered gum canals, which occur as solitary or in groups of 2–4(5), paratracheal parenchyma vasicentric, apotracheal parenchyma scanty, as few diffuse cells among fibres and in the form of short tangential bands enclosing gum canals, 1–6 (mostly 3–5) seriate, distinctly heterocellular rays. The combination of all these characters indicates that the fossil belongs to genus *Dipterocarpus* Gaertn. f. of the family Dipterocarpaceae. From the survey of wood slides and literature, it is found that the fossil shows close resemblance to the wood structure of *Dipterocarpus indicus* Bedd. (BSIP wood slide no. 308) and *D. lowii* Hook. f. (BSIP wood slide no. 2106) as diffuse to diffuse-in-aggregate parenchyma is almost absent in these two species. However, in other wood samples of *Dipterocarpus indicus* amount of parenchyma is much more.

The genus *Dipterocarpus* Gaertn.f. includes about 69 species which are mainly confined to Indo-Malaysian region with maximum development in Borneo, Malaysian Peninsula and Sumatra [26]. The genus ranges in its distribution from India in the west to Philippines in the East. In India, it is found in Assam, the Andamans and the Western Ghats [17]. *Dipterocarpus indicus*, which resembles the fossil closely, is a large tree found in Western Ghats [24] while *D. 1owii* grows in the Malaysian region. In India, the fossil history of genus *Dipterocarpus* traces back to Lower Miocene, and a large number of fossils of different plant parts are recorded from different Neogene exposures. The genus has occurred on the Kerala coast since Miocene i.e., about 15 million years ago [32], [34], [36].

***Neolamarckia*** sp. cf. ***N. cadamba*** Rubiaceae, Figure E, 1–5 in File S1, Figured Specimen–BSIP Museum No. 40083.

The important characteristics of the present carbonized wood are: presence of mostly small to medium sized vessels which occur either as solitary or in radial multiples of 2–5, medium sized inter-vessel pits, parenchyma both paratracheal and apotracheal; paratracheal scanty, apotracheal diffuse to diffuse-in-aggregate, forming uniseriate lines; heterocellular, 1–3 seriate rays with long uniseriate extensions upright cells and non-septate, thin walled fibres. These features collectively indicate its close affinity with the modern woods of the genus *Neolamarckia cadamba* (Roxb.) Bosser (Synonyms: *Anthocephalus cadamba* (Roxb. Miq.; *Nauclea cadamba* Roxb.) of the family Rubiaceae. However, the controversy whether novem *Neolamarckia* should be used or *Anthocephalus* is to be retained, has not yet died down [37]. The classification and phylogeny of *Nauleaea* based on molecular and morphological data favours maintenance of the genus *Neolamarckia* [38]. The carbonized wood also shows superficial resemblance to the woods of family Sapotaceae as they possess similar type of parenchyma and rays, but can be differentiated by the presence of vasicentric tracheids and heavily tylosed vessels.

Rubiaceae (Coffee family) is the fourth largest angiosperm family in wet tropics with more than 80% arborescent genera [39] but very few also occur in temperate and arctic region [29]. It contains 680 genera and more than 10,200 species [26]. Most of the genera occur in forest under storey where their fruits, leaves etc. provide food resources for animals. The genus *Neolamarckia cadamba* (Roxb.) Bosser is widely distributed from India to Malaysian peninsula, Indonesia, Philippines, New Guinea and Australia [37], [40]. It grows best on deep moist alluvial soil along river banks in tropical moist deciduous and evergreen forests. In India, it occurs in sub-Himalayan tract from Nepal eastwards in Darjeeling, Assam, northern Bihar, Orissa and Eastern Ghats. It is not frequently found in southern region, but reappearing in Kadappah and Karnool. In the West coast, it occurs from North Kanara to Travancore and is also found in Andamans [29].

***Rhizophora*** sp. cf. ***R. mangle*** L., Rhizophoraceae, Figure F, 1–6 in File S1, Figured Specimen–BSIP Museum No. 40082

The description is based on wood samples collected from Adichanalloor wetland (9°52′32″ N–76°42′36″ E) in Kollam.

Presence of small vessels with scalariform perforation plates and scalariform intervessel pits, scanty unilaterally paratracheal parenchyma, heterocellular multiseriate rays and thick walled fibres indicate its affinity with the woods of *Rhizophora*, particularly with *R. mangle* L. of the family Rhizophoraceae.

Family Rhizophoraceae consists of 15 genera, often mangroves and 120 species distributed in tropical regions of specially Old World. The genus *Rhizophora* L. is sometimes collectively called true mangroves. *R. mangle* naturally grows in subtropical and tropical regions of both the northern and southern hemispheres between 28° N to S latitudes where they exist in conditions of high salinity, extreme tides, strong winds, high temperatures and muddy and anaerobic soils [41]. The oldest record of *Rhizophora* in India dates back to Oligocene of Assam [42].

## Discussion

### Taphonomy of fossil woods

Though there have been a few reports of fossil woods and partially carbonized logs, these paleosources have not attracted any scientific interest. The reported information is devoid of any description of the geological settings and taphonomy associated with them. Except a few radiocarbon dates, no details are available though they form potential archives of environmental changes and palaeoclimatic signatures. In fact, large and small trunks of trees, often with their bark, parts of the roots and branches intact, are very common in the seasonal and perennial wetlands. In some cases, the wood is partially carbonized especially in the Late Pleistocene sequences, whereas, the Holocene logs are generally hard and are usually found with the bark intact (Figure 3 a–b & e–f). These fossils and sub-fossil logs are regularly retrieved, when the local inhabitants make trenches and canals to irrigate their paddy crop during the dry season, and recover brick clay during mining. Further, the wood parts frequently encounter at intervals while retrieving the subsurface sediments with the help of Standard Penetration Test (SPT) coring. A major part of the wetlands, including the areas associated with the backwaters of Kerala, is referred to as “Karinilam”. Karinalam literally implies “black land”, which is a reference to the high content of organic carbon sediment found in this area. The area is, thereby, one of the most prominent carbon sinks in this part of Peninsular India. In fact, the wetlands and associated landforms hold immense potential for carbon sequestration, and mitigating the impact of climate change due to Global warming in the region. Therefore, the study of terrestrial organic matter particularly that of the buried and preserved woods is significant, while ascertaining the response of vegetation to climate dynamics over a period of time.

Since the region has been subjected to tectonic and hydrologic regimes during the Late Quaternary, the spatio-temporal distribution and environmental setting of these woods are brought to light, while addressing the response of the vegetation to the coastal dynamics. As the fossil logs are found in abundance and are incorporated into peat or lignite (coalified in varying degrees), the cellular details would be preserved in them, and they could be assigned to their “nearest-living-relative” (NRL) or modern analogues. With the aid of the preservation potential of these fossil logs, the growth ring studies may also be carried out as if they were modern woods in dendroclimatology. Accordingly, these ancient woods represent an important source of palaeoclimatic information, which is now being utilized as a proxy. Although, conifers and dicotyledonous woods in temperate and sub-alpine zones have been utilized for climatic reconstruction due to the presence of distinct growth rings and vessels of special arrangement patterns associated with thermal seasonality, tropical and subtropical trees with the exception of Teak (*Tectona grandis*) have seldom been tested. This paucity can be attributed to the lack of information on datable tree-rings of fossil, and modern woods from Indian subcontinent. In coastal areas like Kerala, variation in temperature and humidity is not so evident (non-seasonality). However, Worbes [43], [44] confirmed that in tropical regions, where an annual drought or flood occurs, the wood also exhibits annual growth rings, and the width of growth rings depend on the length of time a tree remains exposed to hydrological extremes. Our efforts over the past few years, persistent excavation, and meticulous search of these fossil woods and sub-fossil logs in the wetlands of Kerala have led to identification of about twenty five species, mainly from tropical evergreen forests, including a few mangrove taxa. These species possess the potential to be employed for dendroclimatic study. The natural affinity of these fossils has been established from anatomical details and ethnobotanical information including local/vernacular names provided by the local inhabitants, who frequently use them for making implements, and fuel due to their high calorific value. Some of these species have already been reported from the Neogene sediments (Mio-Pliocene) that belong to Warkalli Formation, and a few species are still grown, particularly in the sacred grooves, as relict representatives of the pristine forest that had thrived profusely until the Late Pleistocene - Early Holocene in the low lands of Kerala (Figure 3k; Table 1). Although the potential of a few taxa for climate reconstruction has been established, there has hardly been any attempt to utilize them in the paleoclimatic perspective. The few reports in recent years on the buried fossil logs in the wetlands and their utility in environment and culture appraisal, have been sporadic and few [5], [13], [33], [45], [46]. Cherian et al. brought to light recently the practice of usage of local timbers for wharf and boat making in the historical contacts with Mediterranean, Red Sea and Indian Ocean rims [47].

### Chronology of fossil woods

**Figure 5:**
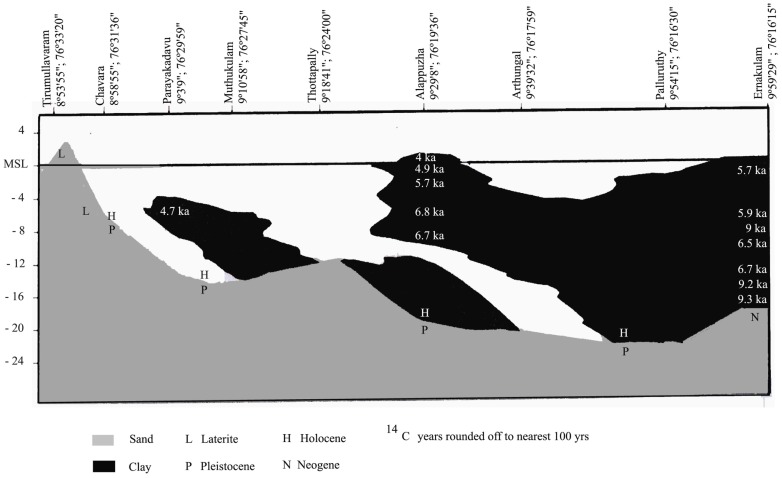
Cross sectional view Pleistocene and Holocene sediments along Kollam - Ernakulam coast.

**Figure 6:**
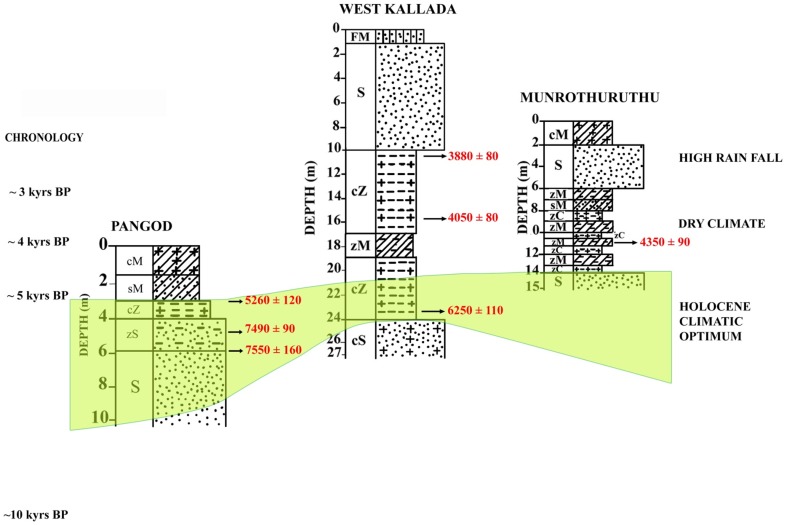
Correlation of Pangod, West Kallada and Munrothuruthu areas showing Holocene Climatic phases.

The studied wood samples have yielded ages that fall mainly into two groups. Wood samples that occur very frequently have yielded ages ranging from *ca* 9000 to 5000 yrs BP (Table 4). This time interval is unique to the entire area, as it falls along the path of South West (SW) Monsoon or Asian Summer Monsoon. The monsoon is stated to have been considerably stronger than the present [1], [5], [48], corresponding to the Holocene Climate Optimum (HCO). The terrain under present investigation now receives 200–400 cm of annual rainfall from the SW Monsoon. This supports the thick tropical rainforest vegetation covering >35% of the states area in the highland - midland and lowland regions. The coastal plains have historically been lush with the mangroves and freshwater swamps and marshes. However, since the late forties this picture has dramatically changed, and the state now has a net forest cover of 15–20% which is fast depleting. Mangroves and other swamps and marshes are confined to less than 10% of their area of that of pre-50 s. If such a thick forest cover can be supported by the present rainfall, it is not difficult to visualize the amount of vegetation that would have been flourishing when rainfall was 2–3 times more than the present. Another sample of wood has yielded an age of *ca* 13000 yrs BP [13].The period 13000–12000 yrs BP is believed to have witnessed intensified monsoon activity. As compared to the Early to Middle Holocene, the terrestrial organic influx during the Late Holocene was considerably lesser. This event is well etched in most of the wetland sedimentary sequences, and is represented by a layer with very little microfloral content and few subfossil woods. The period around 2.5 k yrs BP is considered to have witnessed decreased rainfall, and aridity over large areas of Asian Summer Monsoon. The lower terrestrial organic matter input is attributed to reduced rainfall - a consequence of weak monsoon over the period. The reduced river water influx and the sediment supply to the wetlands have had a significant impact on the preservation of vegetal matter. Considerable areas and sediments of the wetlands have been exposed to subaerial oxidation by 2.5 k yrs BP. The accompanied ecological shift, and the subsequent environmental set up dominated by silty-sand/sand sequence that hardly favored deposition and preservation of tree trunks and organic matter to the extent of Early-Middle Holocene. However, this event is followed by restoration of the monsoon to the present level, but not to the extent of 8000–5000 yrs BP monsoon peak. A generalized cross section distinguishes the Pleistocene and Holocene sediments that entomb the wood remains along Kollam - Ernakulam coast (Figure 5). Palynological and palynodebris analysis of Pangod, West Kallada and Munrothuruthu areas too compliment the palaeoclimate signatures of fossil woods that show the Holocene Climatic phases (Figure 6).

**Table 4:** Radiocarbon dates of the Holocene woods/peat/sediments of the wetlands of Kerala.

SI. No.	Borehole/Sample location	Material dated	Depth (m)	^14^C date yr BP	Median age cal yr BC age 2 sigma	Confidence Interval (2σ, p = 0.954) error 2 sigma	References
1.	Pangod	Wood	3.0	5260±120	4010	120	[13]
2.	Pangod	Wood	5.0	7490±90	6350	90	[13]
3.	Pangod	Sediment	5.0	7480±80	6430	80	[13]
4.	Pangod	Sediment	6.0	7550±160	6380	160	[13]
5.	Meenachil River North	Wood	5.0	2888±78	1100	80	[62]
6.	Meenachil River South	Wood	2.5	5570±30	4400	30	[65]
7.	Meenachil River South	Wood	2.5	5780±95	4650	90	[65]
8.	Meenadom Ar (Meenachil River)	Wood	9.0	6280	-	-	[45]
9.	Mallappally, Manimala basin	Wood	0.3	2820±100	1030	100	[90]
10.	Mallappally, Manimala basin	Wood	0.2	2790±70	970	70	[90]
11.	Paduthodu, Manimala basin	Wood	0.5	3160±70	1410	70	[90]
12.	Komalam, Manimala basin	Wood	9.0	2930±110	1150	110	[90]
13.	Ayirur, Ayirur basin	Wood	2.5	2430±100	580	100	[91]
14.	Udayanapuram, Eastern Periphery of Vembanad lake	Wood	6.0	3810±80	2250	80	PC - Dr. Maya
15.	Elangikkal, Alappuzha District	Wood	1.5	1840±70	190 AD	70	[13]
16.	Pandalam, Achankovil basin	Wood	2.0	5560±90	4420	90	[13]
17.	Vamanapuram River	Wood	-	2910±170	1150	170	[13]
18.	Karamana River	Wood	-	6140±80	5100	80	[13]
19.	Vamanapuram River	Wood	2.0	10540±110	10420	110	[13]
20.	Killiyar, a tributary of Karamana River, Killi	Wood	-	2480±100	600	100	[13]
21.	Karipuzha, Alappuzha district	Wood	2.0	7140±90	6030	90	[13]
22.	Vettiyar, Alappuzha district	Wood	2.0	13880±200	15120	200	[13]
23.	Pathiyoor	Wood	1.0	7510±100	6390	100	[5]
24.	Thannisseri (Iringalakuda)	Peat	2.0	6420±120	5410	120	[5]
25.	Thalasseri	Peat	2.0	7230±120	6130	120	[92]
26.	Poovathumkadavu	Peat	6.5	6720±70	5620	70	[93]
27.	Poovathumkadavu	Peat	24.00	7450±110	6270	110	[93]
28.	Valoor	Peat	2.0	3390±110	1690	110	[93]
29.	Valoor	Peat	3.0	5520±160	4370	160	[93]
30.	Willington Island	Peat	16.75	8315±125	7320	130	[94]
31.	Changa ((Aruvikkara)	Peat	2.5	3300±90	1590	90	[86]
32.	Annallur	Peat	4.0	6630±120	5540	120	[95]
33.	Vembanad Kottayam	Wood	1.0	7050±130	-	-	[96]
34.	Muthukulam, BH2	Wood	12.7–13.5	3660±11	2050	110	[68]
35.	Muthukulam, BH2	Wood	20.7–21.2	6280±110	5230	110	[68]
36.	Muthukulam, BH2	Wood	30.0–31.0	7180±80	6060	80	[68]

### Fossil logs and vegetation types

Of the twenty seven investigated wood samples, twenty five were assigned to their natural taxa based on anatomical features of the nearest relative living form/comparable modern analogues and the ethnobotanical information provided by the local inhabitants (Table 3). Two of them could not be assigned to their NRL due to poor preservation. However, they were found to be dicotyledonous woods. Leaving aside three species of the mangrove, the rest of them belong mostly to the tropical wet evergreen forest types comprising *Artocarpus*, *Calophyllum, Canarium, Dipterocarpus, Diospyros, Shorea, Toona, Holigarna, Hopea, Mimusops, Neolamarckia, Sarcostigma* and *Terminalia*. This indicates their prevalence occurs as a result of high atmospheric humidity, warm temperature and rainfall in the range of 200–750 cm. Semi-evergreen and moist deciduous forms that require annual rainfall <200 cm are *Careya, Dalbergia, Ptercocarpus* and *Lannea*. The families of Anacardiaceae, Burseraceae, Clusiaceae, Dipterocarpaceae, Ebenaceae, Fabaceae, Icacinaceae, Moraceae and Rubiaceae are well represented in the wood assemblage. The genus *Artocarpus* is ubiquitous in the wetlands of Kerala and three species, viz., *A. hirsutus, A. heterophyllous*, and A. *lacucha* have been identified. Investigations have pointed out that the drowned boat excavated by archeologists at Thaikkal was constructed from the wood of *A. hirsutus*, which is endemic to Western Ghats, and is still grown along the Kerala coast [47], [49]. *Dipterocarpus* and *Hopea* of the Dipterocarpaceae and *Calophyllum* of the Clusiaceae also recur often in the studied wood samples. The other significant evergreen taxa are *Canarium*, *Holigarna, Dalbergia* and *Diospyros* (endemic to Western Ghats from Central and South Sahyadri). Many of these taxa have also been recorded in the Neogene sediments of southwestern India, suggesting therein that humid climate and high rainfall prevailed largely during the Early to Middle Holocene period. However, the disappearance of some Dipterocarps like *Anisoptera*, *Dryobalanops* as well as certain species of *Shorea*, *Hopea*, and other evergreen taxa along Kerala coast in the modern flora reflect the response and sensitiveness of these taxa to reduced rainfall due to weak monsoon system since Neogene [50]. Species that thrive under relatively less moist conditions (semi-evergreen type and moist deciduous) include *Careya, Lannea, Pterocarpus, Tectona* and *Terminalia*. These usually represent the youngest stratigraphical sequence of the studied wetlands, and may have been transported from hinterland by rivers. The occurrence of tree trunks of *Rhizophora* sp. cf. *R. mangle, Rhizophora mucronata*, and *Sonneratia apetala*,core mangrove species found in the tidal creeks and littoral forests, indicates a proximity to sea and sea level response to vegetation during the Mid-Holocene transgression, and subsequent burial [14], [51].

**Figure 7:**
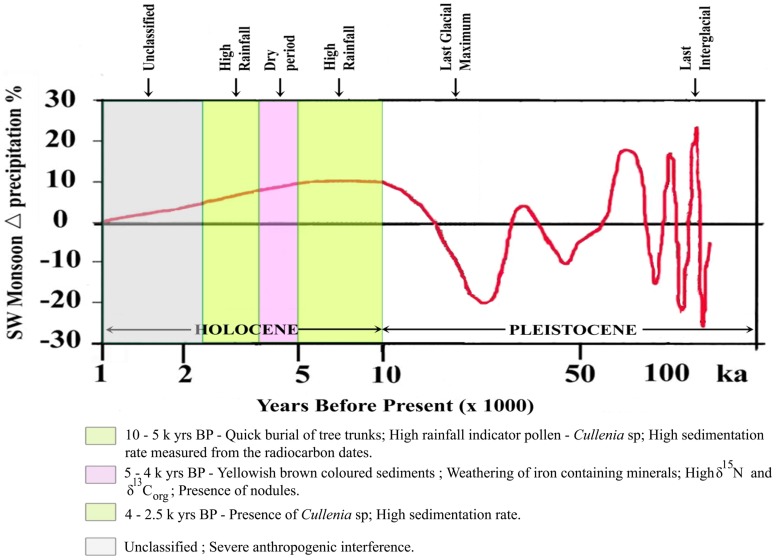
Holocene Climate Model (after [48], [52]).

The taphonomy of woods of the above species at three different intervals: Early Holocene (10.0–6.5 k yrs BP), Middle Holocene (6.5–5.0 k yrs BP) and Late Holocene (<3.6 k yrs BP) has been found to be related to the hydrodynamic regime of the area. This classification emerges in consequence to the impact of the SW and NE monsoon systems where the climatic gradients, rainfall, duration of the dry season and temperature, determine the structural and floristic changes, enabling their distinction into different forest types. The floristic composition of preserved woods of Early to Mid - Holocene indicates a dominance of wet evergreen forest type. The dominance is influenced by the rainfall gradients, as the region is closer to the western slopes of the Western Ghats that are exposed directly to rain-bearing winds of the south-west monsoon. Therefore, the thriving of a dense forest cover during Early to Middle Holocene is mainly attributed to the heavy rainfall. However, it merits emphasis that the present wetlands, where fossil wood and sub fossil logs occur, are not ecologically suited for such a forest cover. It is apparent that such places were not wetlands when the forest cover existed in recent past. The almost catastrophic flood that occurred due to the intensive Monsoon, might have caused the destruction of the thick forest cover, and its subsequent burial in the wetlands. At the beginning of monsoon, intensification around 9000 yrs BP, the sea level was much lower. This would have led to higher stream velocity and hence extensive erosion of river channels and other environments. The sea level rose to the present level around 7000 yrs BP. Subsequently it would have risen further by 2–4 m until about 5000 yrs BP, and then receded to the present level. A sea level higher than that at present, coupled with the excessive rainfall might have paved the way for uprooting the forest vegetation, and burial of the same at its place of growth, or in other sheltered areas further downstream. Besides, the abundant supply of the excessive water that flowed down the streams would supply enough sediments to bury the wood in low lying and flooded land, before its exposure to oxidation by atmosphere, or by shallow water columns. Sediments in any environment can be of geological significance only when they are preserved by subsidence of the environment. In the absence of subsidence, particularly of shallow water bodies, the place of deposition would soon be filled up, and the sedimentary column would be of insignificant thickness. The wetlands and the rivers in the lowland seas subsided in large segments after the wood bearing sediments were deposited. This subsidence was probably accomplished through an eastward tilt of the coast that caused the subsidence of the wetlands, and preservation of these sediments with the fossil wood and sub fossil logs. The accrued data has been used to draw a modified version of the Holocene Climate models [48], [52] (Figure 7).

The accumulation of fossil woods and sub-fossil logs of large forest trees in a large quantity in the South Kerala coastal plains holds immense significance, when the ecology, depositional environment and geological history of the Late Quaternary is inferred. It is likely that extensive floods after the transgression caused massive destruction of the coastal forests, which in turn buried the huge tree trunks. Evidence of such large scale flooding and scouring in the Early Holocene from monsoon Asia has already been dealt with [53]. These fossils are found to be *in situ* accumulation, as roots and branches are found intact in many cases. In some cases even the bark was found intact. This phenomenon constitutes sufficient evidence of a fast burial. Such evidence indicates that these fossils did not drift too far from their place of origin, and hence are of considerable significance in determining vegetation characteristics in the catchments. The profuse growth of wet evergreen, semi-evergreen vegetation comprising many woody species, viz., *Dipterocarpus, Hopea, Shorea, Diospyros, Canarium, Artocarpus, Mangifera, Pterocarpus, Toona, Leea* and *Cullenia* (fossil pollen only) might have been the result of the intensified Asian summer monsoon.

The record of palynoflora and the subfossil logs from the peatland, wetland, palaeoestuaries, river banks and paleobeach ridges from the southwestern part of India undoubtedly prove that the entire land, west of Sahyadri up to the coast, was densely forested towards Early to Mid- Holocene (9.0–5.5 k yrs BP), and this scenario has been attributed to prevailing heavy rainfall [54]. There is a convergence over the view that the beginning of Holocene witnessed an intensification of monsoon which lasted over a few thousand years [55]. During the period from 8.5 to 5.5 k yrs BP, Asia witnessed much higher precipitation, which is now universally accepted as Holocene climatic optimum. The rainfall of this period is often stated to be three times higher than the present rate [56]–[57]. Further, it is interesting to note that this excessive precipitation took place not only in Asia, but also all along the path of summer monsoon. It is during this period that the deserts of Sahara too witnessed development of large lakes and prolific forest cover [58] and the Chad Lake in Central Africa expanded to three times its present state [59]. Therefore, the high rainfall during the Early Holocene was congenial for luxuriant forestation over the entire stretch of land from Sahyadri to the present coast and even beyond the coast in Peninsular India. Prior to 7000 yrs BP, there were hardly any wetlands, except isolated depressions and lakes, while the sea level was much lower [60], [61]. Even the present backwater systems of Vembanad have their origin during the regressive phases (5.0 k–3.0 k yrs BP) of the Holocene [62]. In fact, the high rainfall coupled with a rising sea level must have inundated >75% of the coastal plains until 6.0 k yrs BP. The excessive rainfall, higher than the present rate, was responsible for the development of thick forest and forest swamp cover virtually all over the terrain, including the coastal plains, midland and highlands. The forest cover seems to have extended even up to the present coastline towards Mid-Holocene [13], [46].

The excessive rainfall could have caused some inundation of the low-lying lands. The effect of this flooding was compounded by the sea level rise during the 7.0–6.0 k yrs BP. This converted 75% of the coastal plain land to a veritable lagoon - lake system; this was virtually an abrupt termination of the forest ecosystem. The lower energy level in these newly developed wetlands paved the way for the burial of the hard wooded trees, and decay and disintegration of other forest vegetation in various sediments. However, the condition of preserved wood depends on the type of sediment, and the time lag between the falling of the trees and the time of complete burial. Clay-rich sediment preserves wood and other vegetal remains better as compared to sands and other coarser sediment types. Since the sediment supply was copious due to the high rate of erosion of uplands, the hard wooded trees were entombed in various strata. The woods of relatively softer species must have decayed and became a part of the normal sediment. This is the reason for the high carbon content in the black/grey clay - silty clay sediments that preserves the wood in all the major wetlands of southwestern India.

### Palaeoecological and palaeoclimate potential of fossil logs

During the last glacial event of about 18,000 yrs BP, the Arabian Sea experienced a lowering of its level to the tune of 100–120 m. The consequences of this glacial event have been of immense significance to coastal dynamics, as the landforms responded to a considerable extent such that it caused a submergence of forested areas. It also led to the development of major wetland systems including the ‘Ramsar sites’ like Ashtamudi Lake, Sasthamkotta Lake and Vembanad Lagoon [13], [60]. The occurrence of subfossil logs and carbonized woods in the sediments associated with the wetlands and river bank deposits, demonstrates clearly that the forest vegetation succumbed almost entirely to the coastal dynamics of the Early Holocene (9–7.5 k yrs BP), such that a major part of vegetation cover was lost. The rivers became much more erosive in nature due to the increased gradient, and therefore caused widespread removal of the sediments already deposited, except in inland lakes and depressions. Also, vast areas along the marine shelf got exposed to sub-aerial action. From this lower level, the sea rose in stages and reached the present stage about 7000 yrs BP. The sea level further rose by about 4–6 m inundating the low-lying areas along the coast. This marine inundation was short-lived, and the sea withdrew in stages leaving behind the lagoons and wetlands, and the typical beach deposits even far inland [63]. The coast was re-established at the present place about 3500–4000 yrs BP. The record of this marine inundation is preserved in the landward extension of the offshore basin since a major part of it was subsiding. These movements are found to be at the rate of less than a millimeter to several millimeters per year and have influenced the sedimentary environments to a considerable extent [13]. Therefore, all these factors acting in tandem have produced a complex combination of factors having their effects on ecology and shaping the landforms.

**Figure 8:**
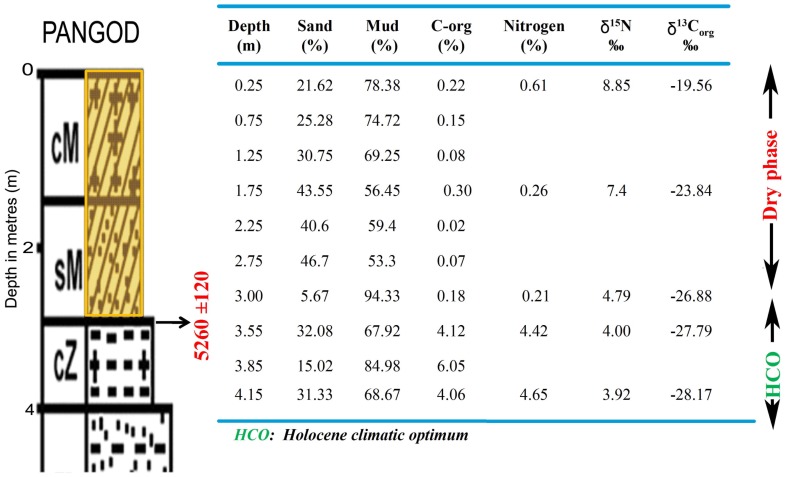
Sand, mud, organic carbon and nitrogen contents in the sediments of Pangod quarry along with the concentration of δ^15^ N and δ ^13^C _org_.

**Figure 9:**
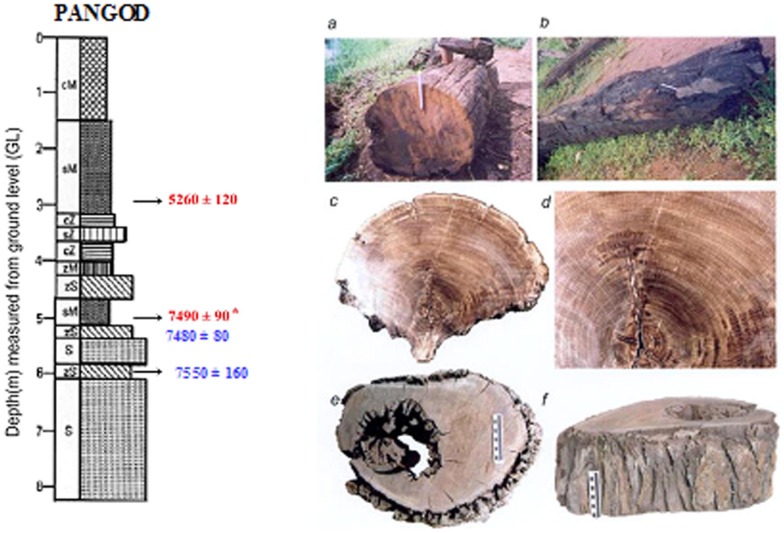
Litholog of Pangod quarry showing carbonized and non carbonized subfossil logs at two different stratigraphic levels along with radiocarbon dates. (a) Sub fossil log. (b) Partially carbonized wood. (c) Cross section showing indistinct growth rings. (d) Enlarged sector of growth rings. (e) and (f) Carbonized wood along with bark showing disintegration of wood.

The area of investigation presently receives annual rainfall ranging from 200 to >500 cm; about 70% of it is from the SW monsoon that is known as the Asian Summer Monsoon. Palynological data of the Kerala coast [63]–[65] too complements the accrued wood assemblage for palaeoclimate appraisal. Palynodebris and sediment characteristics suggest that there were two periods of abnormal high rainfall: one before the last glacial maximum (LGM), and another during Early Holocene [63], [64]. Many of the landforms in the coastal plains and several of the landforms in the hinterland are proved to contain partial to complete record of the period from Late Pleistocene to Holocene geo-environmental records [5]. Besides, there are positive indications that some of these terrains were sub-aerially exposed lands, which were once thickly forested. As a result of abnormal high rainfall coupled with tectonics, and the antecedent landform characteristics, the trees that grew there were possibly uprooted and buried *in situ*. This is particularly seen in the seasonal wetlands peripheral to the two main lagoonal water bodies namely the Vembanad and Kayamkulam lagoons [60], [64]. Further, the sediments in the freshwater lakes and abandoned river channels as well as in many river terraces contain tree trunks and carbonized wood. In many cases, the trees were buried under variable thickness of sediments before being destroyed by atmospheric processes due to the continued influx of terrigenous sediments under the transgressive phases of the sea. The events are well reflected in the δ^13^C_org_ and δ^15^N values estimated for the Pangod borehole core (Figure 8). Though, the upper yellowish brown mud rich layer records low organic carbon content (0.12%) compared to the underlying carbonaceous clay (6.05%), the δ^13^C_org_ exhibits an opposite trend [63]. This clearly indicates a gradual change in the depositional regime from terrestrial (δ^13^C −28.17‰) to marine entity (−19.56‰). The sediments of marine origin generally contain higher δ^13^C_org_ values, as a major part of it is derived from marine phytoplankton with higher δ^13^C_org_ values [66], [67]. The ^14^C age of upper most part of the organic carbon rich layer, just below the yellowish brown silt and clay layer at 3 m bgl is ^14^C dated as 5260±120 yrs BP. The low δ^13^C_org_ values of −28.17‰ to −26.88‰ show that the organic input in the carbonaceous clay is from C_3_ plants that flourished in the hinterlands during Early Holocene in Pangod section (Figure 9). The δ^15^N values vary from 3.92‰ to 8.85‰, with highest values recorded for the top yellowish brown layer [63]. The enhanced level of δ^15^ N isotope in the surface sediments as compared to the lower organic rich layers points to degradation and preferential consumption of lighter isotopes, and subsequent enrichment of heavier δ^15^N, a feature revealed elsewhere earlier [67]. Comparatively lower δ^15^N values in the layer also points to the preservation potential of organic matter, derived from terrigenous sources possibly aided by abnormal rainfall as recorded in Muthukulam core of SKSB during the Early to Mid-Holocene period [68].

**Figure 10:**
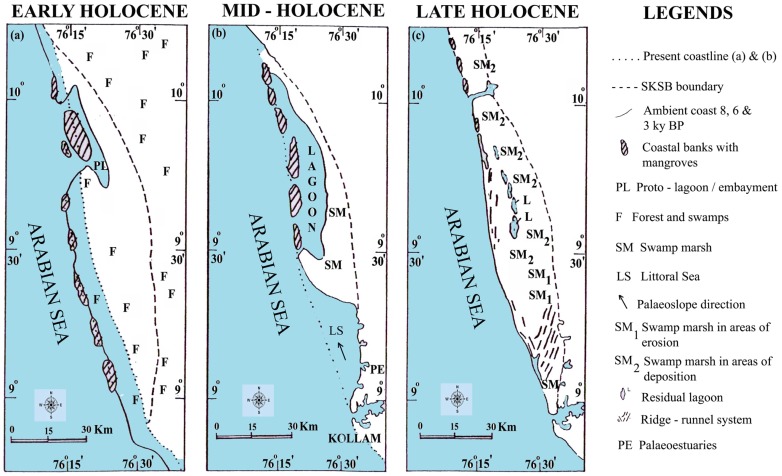
Ecogeomorpholgical evolution of southwest coast of India during Holocene. (a) 9–8 k yrs BP; (b) 7–6 k yrs BP; (c) 3 k yrs BP.

The area of investigation had landforms supporting dense forest vegetation prior to Holocene transgression of sea, part of which could be preserved in suitable environments. This is a factor unique to southwestern coast of India and as such it has significance while addressing the palaeoclimate potential of the past forest remains. The proposed eco-geomorphological model displays the modifications of the coastline and associated landforms and forests since Early Holocene (Figure 10). However, the forest vegetation preserved is an infinitesimally small part of the one that thrived, but constitutes a major terrestrial palaeoclimate proxy from the Indian subcontinent. The evidence of plant macrofossil archive in the form of buried forests for Mid-Holocene Thermal Maximum in southwestern India is consistent with earlier reports on intensified and prolonged Asian Monsoon from other parts of west coast of India [55], [64], [65], [69]–[77], the Deccan Trap region [78], the Nilgiri hills in south India [57], [79], Ganga Plains [80], [81], Southern Oman [82], the northwestern Pacific [83] and the Indus delta [84]. In addition, there are numerous locales that were favorable for development of fresh water swamps and marshes and lagoonal and marginal marine mangrove swamps, which have been eventually converted into land in the recent past.

## Conclusions

Considering the scarcity of data for Holocene climate variability from lower latitudes, the accrued plant macrofossil and pollen proxies from the sedimentary archives of southwest India imply a Mid-Holocene Thermal Maximum related to intensified Asian Summer Monsoon, as recorded elsewhere. The degree of preservation and relative abundance of the wood remains varies considerably. The variation is dependent mainly on the lithological characteristics of the landform units, and the associated hydrological regimes under which they are deposited. All the fossil wood and sub-fossil logs retrieved, though insufficient for generalization, have yielded ages prior to the Holocene transgression (7.0–6.5 k yrs BP). Evidence suggests that the entire terrain west of Sahyadri (Western Ghats) was thickly forested during the Holocene climatic optimum (9.0–6.0 k yrs BP), when the region had witnessed a spell of heavy precipitation, ∼2–3 times more than the present. The flooding of the forest probably occurred as a result of intensified and prolonged Indian Summer Monsoon coupled with sea level rise to the present level or slightly above. This led to a drastic increase in the sluggishness of the river flows that resulted in abrupt flooding of the forest habitat, where the trees thrived. The increased fluvial sediment supply by the rivers enabled some of the tress to be buried and preserved as such, even before the formation of the major backwater systems including the Vembanad Lagoon. Further, the present ecology is unsuitable to support evergreen forests. It can be concluded then that coastal plains and associated landforms were covered by thick tropical evergreen forests, which got destroyed by flooding towards Middle Holocene. Though the plant macrofossil record represents a fraction in terms of taxa of the wet evergreen forests, their relative abundance along with dissolved carbon makes the wetlands and associated landforms one of the best carbon sinks in India. Besides acting as a carbon sink, the wetlands can also serve as a source of carbon, in that they may supply significant amounts of carbon to adjacent coastal ecosystems. This in turn, plays a vital role in coastal dynamics and overall productivity of the region. Thus, the wetlands of southwest India hold immense potential for palaeoecological reconstruction of long-term landscape, and vegetation changes.

## Supporting Information

File S1 **Supporting Information.** Text S1: Systematic description of sub fossil logs. Figure A: Anatomical details of *Artocarpus* sp. cf. *A. lacucha* Buch-Ham. Figure B: Anatomical details of *Careya arborea* Roxb. Figure C: Anatomical details of *Diospyros* sp. cf. *D. bourdilloni* Brandis. Figure D: Anatomical details of *Dipterocarpus* sp. cf. *D. indicus* Teysm. ex Miq. Figure E: Anatomical details of *Neolamarckia* sp. cf. *N*. *Cadamba*. Figure F: Anatomical details of *Rhizophora* sp. cf. *R. mangle* L.
(ZIP)
