# Correction: Vegetation Response and Landscape Dynamics of Indian Summer Monsoon Variations during Holocene: An Eco-Geomorphological Appraisal of Tropical Evergreen Forest Subfossil Logs

**DOI:** 10.1371/journal.pone.0109455

**Published:** 2014-09-23

**Authors:** The PLOS ONE Staff

There is an error in the beginning of the second paragraph of the “Systematic description of identified woods” sub-section of the Observations and Results. The correct sentence is: Artocarpus sp. cf. A. lacucha Buch-Ham., Moraceae, Figure S1, 1-5 in File S1; Figured Specimen – BSIP Museum No. 40081.

There is an error in reference 91. The correct reference is: Rajendran CP, Rajagopalan G, Narayanaswamy (1989) Quaternary geology of Kerala: evidence from radiocarbon dates. Journal of Geological Society of India 33: 218 - 222.

There is an error in the legend for Figure 1. Please see the corrected Figure 1 here.

**Figure 1:**
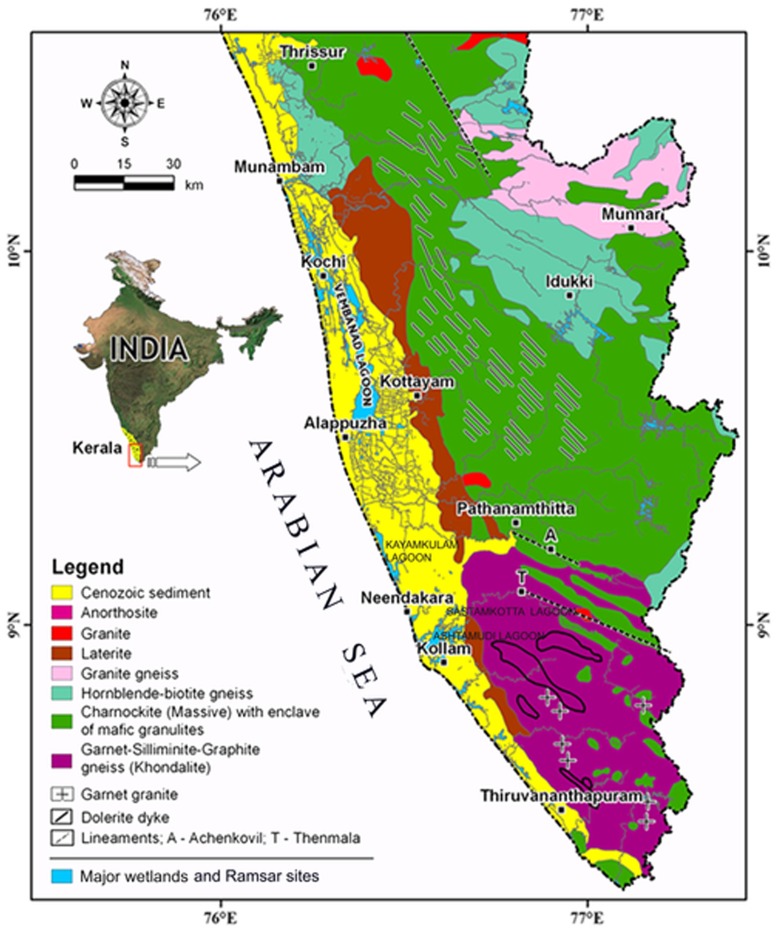
Location of major wetlands in Southwestern India showing geological formations/rock type and lineaments (Modified after [96]).

## References

[1] Kumaran NKP, Padmalal D, Nair MK, Limaye RB, Guleria JS, et al (2014) Vegetation Response and Landscape Dynamics of Indian Summer Monsoon Variations during Holocene: An Eco-Geomorphological Appraisal of Tropical Evergreen Forest Subfossil Logs. PLoS ONE 9(4): e93596 doi:10.1371/journal.pone.0093596 24727672 10.1371/journal.pone.0093596 PMC3984104

