# Supplementary material for: Vegetation Response and Landscape Dynamics of Indian Summer Monsoon Variations during Holocene: An Eco-Geomorphological Appraisal of Tropical Evergreen Forest Subfossil Logs
Source: PLoS One. 2014 Apr 11;9(4):e93596. doi: 10.1371/journal.pone.0093596 (PMC3984104; doi:10.1371/journal.pone.0093596)
Supplement: File S1 — Supporting Information. Text S1: Systematic description of sub fossil logs. Figure A: Anatomical details of Artocarpus sp. cf. A. lacucha Buch-Ham. Figure B: Anatomical details of Careya arborea Roxb. Figure C: Anatomical details of Diospyros sp. cf. D. bourdilloni Brandis. Figure D: Anatomical details of Dipterocarpus sp. cf. D. indicus Teysm. ex Miq. Figure E: Anatomical details of Neolamarckia sp. cf. N. Cadamba. Figure F: Anatomical details of Rhizophora sp. cf. R. mangle L. (ZIP) [file pone.0093596.s001.zip › File S1 24.3.14/Text S1 Systematic description 21.3.14.docx]

SYSTEMATIC DESCRIPTION

**Family- Moraceae**

Tribe- Artocarpeae

Genus –*Artocarpus* Forster J. R. and Forster G.

*Artocarpus* sp. cf. *A. lacucha* Buch-Ham.

(Figure A in File S1 , 1-5)

## The specimen is well preserved showing all the anatomical details.

**Description-** *Wood* diffuse porous. *Growth rings* not seen. *Vessels* medium to large, tangential diameter 150-230 µm, radial diameter 240-345 µm, solitary to radial multiples of 2-3, heavily tylosed, circular to oval in shape(Figure A, 1), 6-9 per sq mm; vessel members 232- 472 µm long with transverse to oblique end walls, perforations simple; intervessel pits alternate, bordered, 8-10 µm in diameter (Figure A, 5). *Axial parenchyma* paratracheal, vasicentric, 5-8 cells around the vessels, sometimes aliform to confluent forming 4-8 thick bands at places (Figure A, 1, 4), cells thin walled, 22-42 µm in diameter and 136-145 µm long. *Rays* 1-5 seriate, heterocellular (Figure A, 3), uniseriate made up of both procumbent and upright cells, 12-20 μm in width and 3-9 cells or 112-360 μm in length, multiseriate made up of procumbent cells in the median portion with 1-3 marginal rows of upright cells at one or both the ends (Figure A, 2), 19-50 μm in width and 6-30 cells or 110-600 μm in length, ray to ray fusion observed; upright cells 33-56 μm in tangential height and procumbent cells 11-25 μm in tangential height. *Latex tubes* radial, present in multiseriate rays, small, single per ray, 35-50 m in diameter; epithelial lining not preserved due to rupturing of cells (Figure A, 2). *Fibres* non-libriform, non-septate, 14-18 μm in diameter and 650-1360 μm in length.

*Figured Specimen –* BSIP Museum No. 40081

Family –Clusiaceae

**Genus – *Calophyllum*** Linn.

***Calophyllum* sp.**

***Description* -** *Wood* diffuse – porous. *Growth rings* not seen. *Vessels* almost exclusively solitary, sometimes closely spaced, arranged in oblique radial lines (Figure 4, 1); 8-10 per sq. mm; small to medium sized, tangential diameter 55-170µm, radial diameter 100-220 µm; circular to elliptic; occluded with tyloses; lumen filled with red gummy deposits; vessel members 220-440 µm in length with truncate or oblique end walls; perforations simple; intervessel pits not seen. *Parenchyma* abundant, apotracheal, forming 6-10 seriate broken bands among fibres (Figure 4, 1); parenchyma cells 11-33 µm in diameter and 44-66µm in length, crystalliferous, single large crystal frequently present. *Rays* uniseriate, occasionally biseriate due to pairing of cells in median portion, closely spaced, 30-40 rays per mm (Figure 4, 2-3); heterocellular made up of both upright and procumbent cells (Figure 4, 3-4); 2-20 cells or 35-440 µm long; single large crystal present in ray cells (Figure 4, 3); procumbent cells with tangential height 11-22 µm and radial length 44-77 µm; upright cells 33-55 µm in tangential height and 22-33 µm in radial length. *Fibre tracheids* aligned between two consecutive rays, angular, 11-33 µm in diameter; non septate. *Vasicentric tracheids* present, circular to oval in cross section, forming 2-3 seriate sheaths round the vessels, pits present in vertical rows.

*Figured Specimen –* BSIP Museum No. 40078A

#### Family– Lecythidaceae

Subfamily- Planchonioideae

Genus– *Careya* Roxb.

*Careya* *arborea* Roxb.

(Figure B in File S1, 1-6)

**Description -** *Wood* diffuse porous. *Growth rings* inconspicuous. *Vessels* solitary, as well as in radial multiples of 2-4 (sometimes up to 6), sometimes into small clusters (Figure B, 1-2); 8-12 per sq. mm; small to medium sized, tangential diameter 50-183 µm, radial diameter 96-182 µm; circular to oval when solitary, flattened at the place of contact when in multiples (Figure B, 5-6); occluded with tyloses; vessel members 330 - 458 µm in length with oblique end walls; perforations simple; intervessel pits alternate, bordered, orbicular to oval, about 8.5-11 µm in diameter with linear horizontal apertures (Figure B, 6) . *Axial parenchyma* abundant, both apotracheal and paratracheal; paratracheal scanty, vasicentric forming 1-2 seriate sheath round the vessels, sometimes sparse, few cells confined to tangential walls of the vessels; apotracheal abundant, diffuse to diffuse-in-aggregate forming 1-2 seriate broken lines among fibres forming reticulum (Figure B,1-2); parenchyma cells 33-55 µm in diameter and 66-110 µm in length. *Rays* 1-4 (rarely up to 6) seriate, 8-12 rays per mm; heterocellular (Figure B, 3-5); uniseriate few, made up of upright cells only, 6-10 cells or 225-550 µm long; multiseriate 2-5 seriate, made up of procumbent cells in the centre with 2-6 marginal row of up right cells at one or both the ends (Figure B, 3-4), , 12-40 cells or 200-800 µm long; end to end ray fusion occasionally present; ray cells crystalliferous (Figure B, 4), procumbent cells with tangential height 12-16 µm and radial length 30-44 µm; upright cells 36-60 µm in tangential height and 12-36 µm in radial length (Figure B, 4-5). *Fibres* aligned between two consecutive rays, angular, 11-33 µm in diameter; non septate.

*Figured Specimen –* BSIP Museum No. 40080

**Family- Ebenaceae**

Genus- *Diospyros* L.

*Diospyros* sp. cf. *D. bourdilloni* Brandis

(Figure C in File S1, 1-7)

**Description:** *Wood* diffuse porous (Figure C, 1). *Growth rings* indistinct, delimited by thick walled fibres. *Vessels* small to medium, solitary or in radial multiples of 2-5 (sometimes 6) (Figure C, 1-2), evenly distributed, 6-12 per sq mm, open, tangential diameter 61-110 µm, radial diameter 103-160 µm, when solitary oval in shape, laterally compressed, vessel elements with tailed ends (Figure C, 4) ; perforations simple; intervessel pits alternate, small, 4-6 µm in size (Figure C, 4). *Axial parenchyma* both apotracheal and paratracheal, paratracheal parenchyma scanty, a few cells around the vessels or sometimes forming a thin complete or incomplete sheath around the vessels, apotracheal parenchyma abundant, diffuse to diffuse-in-aggregate, occurring as interrupted uniseriate concentric lines at regular intervals forming reticulate pattern with the rays (Figure C, 1-2), parenchyma cells thin walled, about 12-16 µm in diameter and 55-64 µm in length. *Rays* fine, mostly uniseriate, biseriate due to pairing of procumbent cells through the central portion are also quite common (Figure C, 3, 5), short or moderately long, 2-16 cells or 85-279 µm long and 15-21 µm wide, closely spaced, heterocellular (Figure C, 6), rays made up of procumbent cells in the middle portion with a tail of upright cells at the ends, single large crystals are abundant in ray cells (Figure C, 3, 5); tangential diameter of procumbent cells 10-20 µm and radial length 30-50 µm, tangential diameter of upright cells 40-63 µm and radial length 25-30 µm; vessel-ray pits many per cell, elongated, borders not seen (Figure C, 6-7). *Fibres* non-libriform, non-septate and 10-12 µm in diameter.

*Figured Specimen –* BSIP Museum No. 40079

**Family: Dipterocarpaceae**

Subfamily: Dipterocarpoideae

Genus: *Dipterocarpus* Gaertner f. 1805

*Dipterocarpus* sp. cf. *D.* *indicus* Bedd.

(Figure D in File S1, 1-5)

**Description**: *Wood* diffuse porous (Figure D, 1). *Growth rings* indistinct. *Vessels* small to large, mostly medium to large, tangential diameter 91-207 μm, radial diameter 114-275 μm, predominantly solitary, rarely in radial multiples, usually round to oval but variously shaped due to compression, evenly distributed, 8-12 per sq mm, heavily tylosed (Figure D,1-2); vessel members truncate with oblique ends, 230-446 μm in height; perforations simple; intervessel pits could not be observed due to heavily tylosed vessels and rare occurrence of paired or radial multiples of vessels. *Tracheids vasicentric* occurring in the immediate vicinity of vessels, intermingled with parenchyma, recognizable in longitudinal section in having bordered pits, pits 6-8 μm in diameter. *Axial parenchyma* both paratracheal and apotracheal; paratracheal vasicentric, sometimes confluent connecting a few adjacent vessels; apotracheal sparse, rarely few cells dispersed among the fibres, also in the form of short tangential bands enclosing gum canals (Figure D, 1-2); cells 9-17 μm in diameter and 65-90 μm in length. *Rays* 1-5 (rarely 6) seriate; uniseriate rays made up of either upright or both procumbent and upright cells, about 16-27 μm in width and 3-7 cells or 136-326 μm in height (Figure D, 3-4); multiseriate rays made up of procumbent cells in the central portion with 1 to several marginal rows of upright cells at one or both the ends (Figure D, 3-4), 29-70 μm in width and 8-30 cells or 268-820 μm in height; sheath cells present on one or both the flanks (Figure D, 3-4); ray to ray fusion observed; ray tissue heterocellular (Figure D, 5), ray cells crystalliferous; procumbent cells 11-26 μm in tangential height; upright cells 33-40 μm in tangential height. *Fibres* angular in cross section, nonseptate, 12-18 μm in diameter and about 1260-1895 μm in length. *Gum canals* normal, vertical, embedded in parenchyma, solitary or in short tangential rows (Figure D, 1-2), smaller than vessels, tangential diameter 31-55 μm, radial diameter 54-98 μm.

*Figured Specimen –* BSIP Museum No. 40078

Family- Rubiaceae

Subfamily- Cinchonoideae

Tribe- Naucleeae

Genus- *Neolamarckia* sp. cf. *N. cadamba*

(Figure E in File S1,1-5)

**Description**- *Growth rings* present, indistinct, demarcated by change in fibre wall thickness and difference in vessel density (less frequency). *Wood* diffuse porous. Vessels solitary and in radial multiples of 2-3 (sometimes up to 5) (Figure E, 1-2), circular to oval when solitary, small to medium tangential diameter 20-74 µm, radial diameter 35-133 µm, 5-25 vessel per sq. mm, vessel elements 350-560 µm in length; perforations simple; intervessel pits vestured, alternate, polygonal, medium, 7-11 µm in diameter (Figure E, 5), tyloses absent. *Axial parenchyma* paratracheal and apotracheal, paratracheal parenchyma scanty, few cells associated with some of the vessels, apotracheal parenchyma diffuse to diffuse-in-aggregate, forming short uniseriate line of 5-16 or more cells (Figure E,1-2), cells 12-15 µm in diameter and 130-156 µm in length. *Rays* heterocellular (Figure E, 3-4), 1-3 seriate, uniseriate made up of upright or square cells only, 7-12 cells or 200-410 µm long, multiseriate rays composed of procumbent cells in the centre with long uniseriate extensions of 2-4 upright or square cells at both the ends (Figure E, 3), 7-23 cells or 165-758 µm long, single crystals present in upright cells, procumbent cells 23-35 µm tangential height and 40-80 µm in radial length, upright cells 45-88 µm and tangential height and 33-56 µm in radial length. Fibres aligned in radial rows, nonlibriform, nonseptate, fibre pits bordered, common in both radial and tangential walls.

*Figured Specimen –* BSIP Museum No. 40083

**Family- Rhizophoraceae**

*Genus-Rhizophora* L.

*Rhizophora* sp. cf. *R. mangle* L.

(Figure F in File S1, 1-6)

**Description-** *Wood* diffuse porous (Figure F, 1-2). *Growth rings* indistinct. *Vessels* small, 26-86 µm in tangential diameter, solitary or in radial multiples of 2-4 (Figure F, 1-2), evenly distributed, 6-20 per sq. mm; perforations plates scalariform, plates with less than 8 bars; intervessel pits scalariform (Figure F, 6); vessel elements 300-380 µm long. *Axial parenchyma* scanty, unilaterally paratracheal, sometimes vasicentric to aliform (Figure F, 1-2), lumina filled with red gum. *Rays* small to large, 10-14 per mm; uniseriate rays rare, mostly 2-4 (occasionally 5) seriate (Figure F, 3-4), more than 100 cells or 251-2,683 µm long and 28-41 µm wide, weakly heterocellular (Figure F, 4), made up of procumbent cells with one to two rows of upright or square cells at one or both the ends, tangential diameter of procumbent cells and upright cells 12-22 µm and 27-32 µm, respectively. *Fibres* very thick walled, libriform, 7-9 µm in diameter, non-septate, frequently pitted, pits simple (Figure F, 3-4)

*Figured Specimen –* BSIP Museum No. 40082

**Figure A in File S1**

*Artocarpus* sp. cf. *A. lacucha* Buch-Ham.

1. Cross section showing nature and distribution of vessels, parenchyma and rays (BSIP Museum Slide No. 40081- 1).
2. Tangential longitudinal section showing fine and broad ray with gum duct marked with arrow (BSIP Museum Slide No. 40081- 2).
3. Radial longitudinal section showing heterocellular nature of rays (BSIP Museum Slide No. 39809- 3).
4. Enlarged cross section showing paratracheal parenchyma (BSIP Museum Slide No. 40081- 1).
5. Large intervessel pits as seen in Tangential longitudinal section (BSIP Museum Slide No. 40081- 2).

**Figure B in File S1**

*Careya* *arborea* Roxb.

1. Cross section showing nature and distribution of vessels, parenchyma and rays (BSIP Museum Slide No. 40080- 1).
2. Enlarged cross section showing tylosed vessels, rays and paratracheal and diffuse in aggregate parenchyma (BSIP Museum Slide No. 40080- 1).
3. Tangential longitudinal section showing multiseriate rays and parenchyma strands marked with arrow (BSIP Museum Slide No. 40080- 2).
4. Tangential longitudinal section showing multiseriate rays with enlarged crystalliferous cells (BSIP Museum Slide No. 40080- 2).
5. Radial longitudinal section showing heterocellular nature of rays with crystal in upright cells marked with arrow (BSIP Museum Slide No. 40080- 3).
6. Intervessel pits as seen in tangential longitudinal section (BSIP Museum Slide No. 40080- 2).

**Figure C in File S1**

*Diospyros* sp. cf. *D. bourdilloni* Brandis

1. Cross section showing nature and distribution of vessels, parenchyma and rays (BSIP Museum Slide No. 40079- 1).
2. Enlarged cross section showing radial multiple of vessels, and distribution of parenchyma (BSIP Museum Slide No. 40079- 1).
3. Tangential longitudinal section showing uniseriate and biseriate rays with crystalliferous cells (BSIP Museum Slide No. 40079- 2).
4. Tangential longitudinal section showing intervessel pits and vessel element with tailed ends (BSIP Museum Slide No. 40079- 2).
5. Tangential longitudinal section showing distribution of rays with crystalliferous cells (BSIP Museum Slide No. 40079- 2).
6. Radial longitudinal section showing heterocellular nature of rays (BSIP Museum Slide No. 40079- 3).
7. Enlarged radial longitudinal section showing elongated vessel-ray pits in (BSIP Museum Slide No. 40079- 139805- 2).

**Figure D in File S1**

*Dipterocarpus* sp. cf. *D.* *indicus* Teysm. ex Miq.

1. Cross section showing nature and distribution of vessels, parenchyma and vertical gum canals marked with arrow (BSIP Museum Slide No. 40078- 1).
2. Same section enlarged showing distribution of vessels filled with tyloses, parenchyma and small gum canals (BSIP Museum Slide No. 40078- 1).
3. Tangential longitudinal section showing heterocellular rays and vessels (BSIP Museum Slide No. 40078- 2).
4. Tangential longitudinal section showing heterocellular rays with sheath cell on the flanks marked with arrow (BSIP Museum Slide No. 40078- 2).
5. Radial longitudinal section showing heterocellular rays, vessels filled with tyloses (BSIP Museum Slide No. 40078- 3)

**Figure E in File S1**

*Neolamarckia* sp. cf. *N. cadamba*

1. Cross section showing nature and distribution of vessels, parenchyma, growth rings marked with arrow and rays (BSIP Museum Slide No. 40083- 1).

2. Enlarged cross section showing distribution of vessels, rays and apotracheal diffuse in aggregate parenchyma lines (BSIP Museum Slide No. 40083- 1).

3. Tangential longitudinal section showing heterocellular rays (BSIP Museum Slide No. 40083- 2).

1. Radial longitudinal section showing heterocellular nature of rays (BSIP Museum Slide No. 40083- 3).
2. Intervessel pits as seen in tangential longitudinal section (BSIP Museum Slide No. 40083- 2).

**Figure F in File S1**

*Rhizophora* sp. cf. *R. mangle* L.

1. Cross section showing nature and distribution of vessels, parenchyma and rays (BSIP

Museum Slide No. 40082- 1).

2. Same section enlarged showing distribution of vessels, rays and unilaterally paratracheal

parenchyma and thick fibres (BSIP Museum Slide No. 40082- 1).

3. Enlarged tangential longitudinal section showing rays with gum ducts and tylosed vessel

(BSIP Museum Slide No. 40082- 2).

4. Tangential longitudinal section showing broad rays and frequently pitted fibres marked

with arrow (BSIP Museum Slide No. 40082- 2).

5. Radial longitudinal section showing heterocellular nature of rays (BSIP Museum Slide No.

40082- 3).

6. Scalariform intervessel pits (BSIP Museum Slide No. 40082- 2).
