# Supplementary figures and images for: Vegetation Response and Landscape Dynamics of Indian Summer Monsoon Variations during Holocene: An Eco-Geomorphological Appraisal of Tropical Evergreen Forest Subfossil Logs
Source: PLoS One. 2014 Apr 11;9(4):e93596. doi: 10.1371/journal.pone.0093596 (PMC3984104; doi:10.1371/journal.pone.0093596)

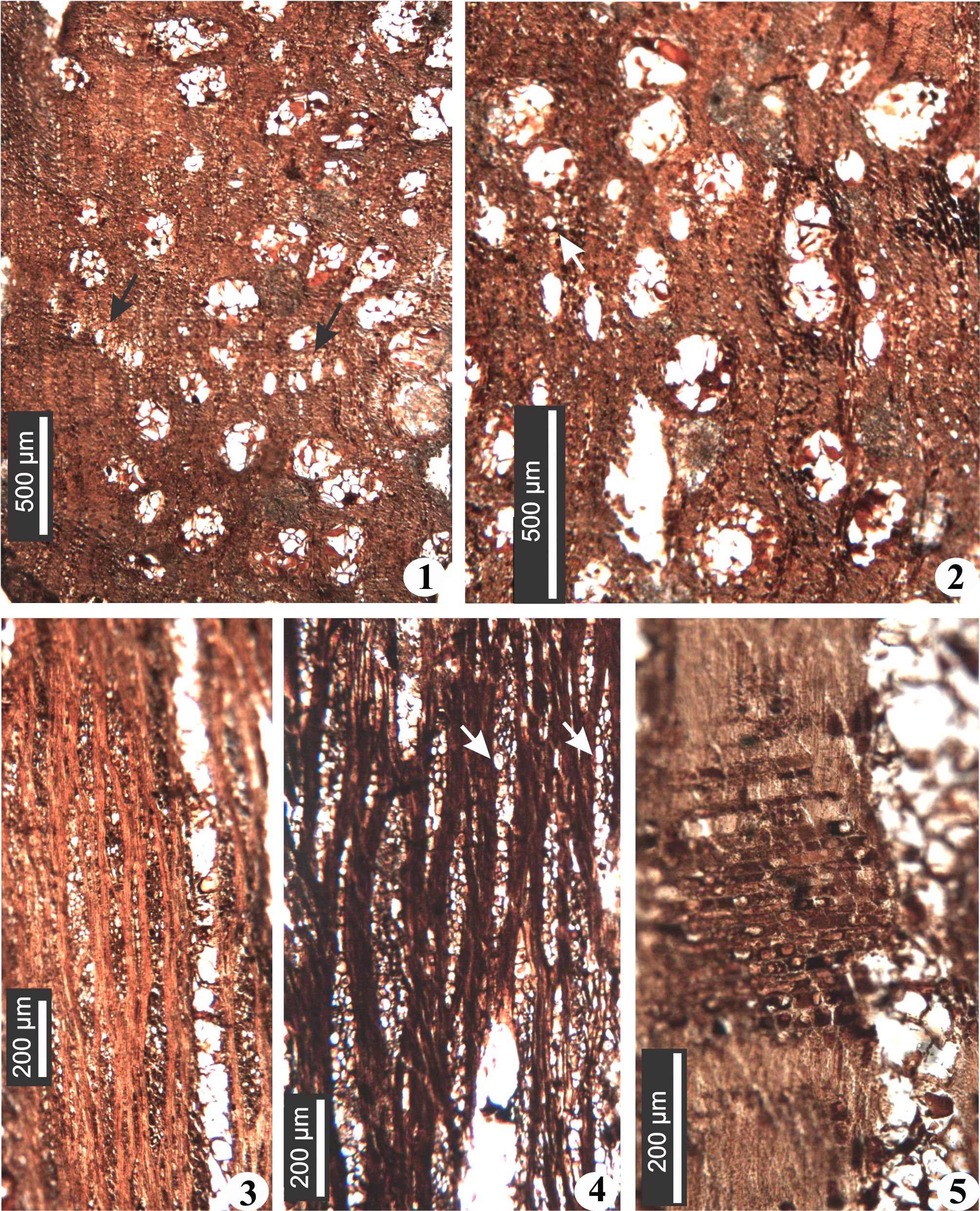

Supplement: File S1 — Supporting Information. Text S1: Systematic description of sub fossil logs. Figure A: Anatomical details of Artocarpus sp. cf. A. lacucha Buch-Ham. Figure B: Anatomical details of Careya arborea Roxb. Figure C: Anatomical details of Diospyros sp. cf. D. bourdilloni Brandis. Figure D: Anatomical details of Dipterocarpus sp. cf. D. indicus Teysm. ex Miq. Figure E: Anatomical details of Neolamarckia sp. cf. N. Cadamba. Figure F: Anatomical details of Rhizophora sp. cf. R. mangle L. (ZIP) [file pone.0093596.s001.zip › File S1 24.3.14/Figure D Dipterocarpus.jpg]

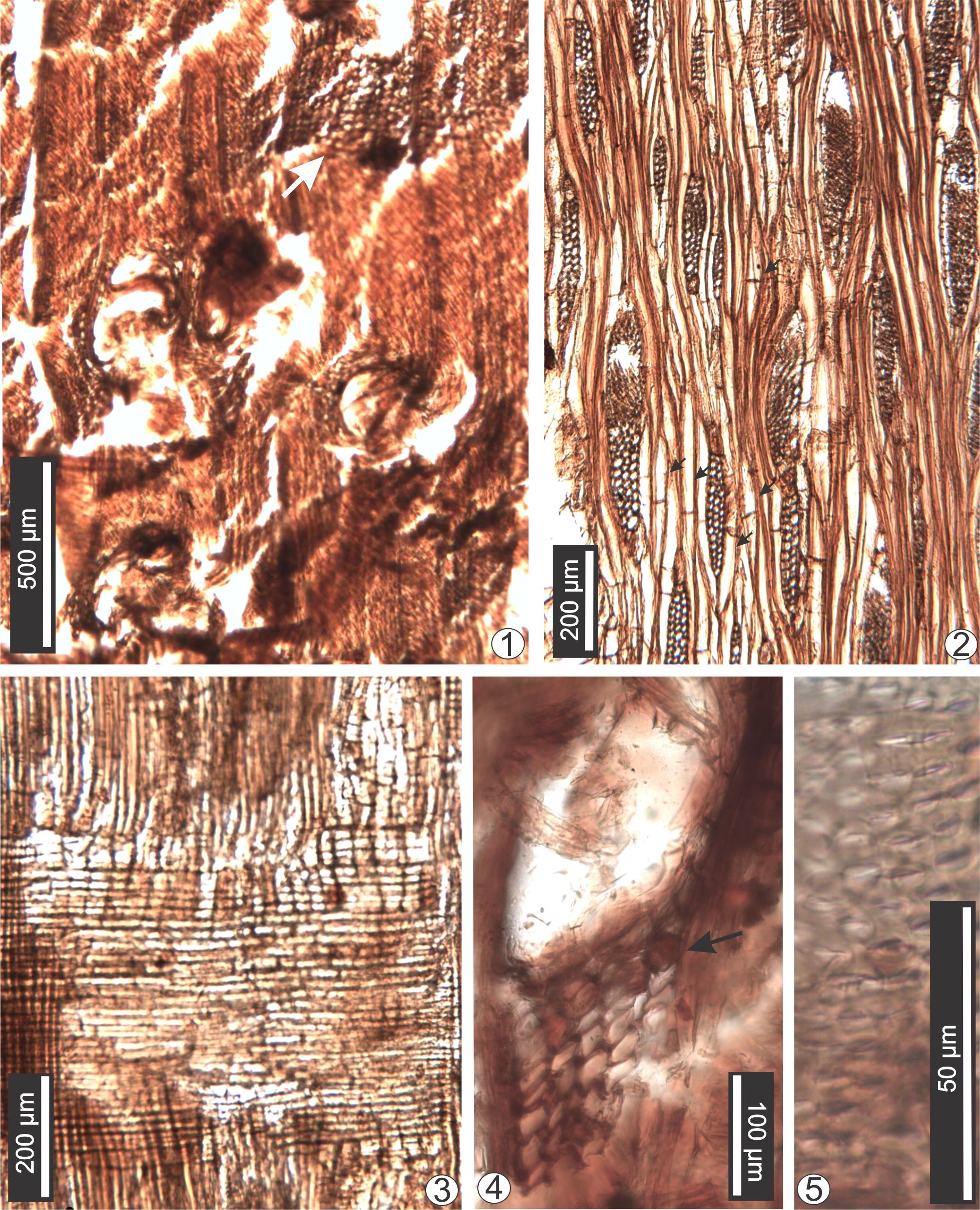

Supplement: File S1 — Supporting Information. Text S1: Systematic description of sub fossil logs. Figure A: Anatomical details of Artocarpus sp. cf. A. lacucha Buch-Ham. Figure B: Anatomical details of Careya arborea Roxb. Figure C: Anatomical details of Diospyros sp. cf. D. bourdilloni Brandis. Figure D: Anatomical details of Dipterocarpus sp. cf. D. indicus Teysm. ex Miq. Figure E: Anatomical details of Neolamarckia sp. cf. N. Cadamba. Figure F: Anatomical details of Rhizophora sp. cf. R. mangle L. (ZIP) [file pone.0093596.s001.zip › File S1 24.3.14/Figure A Artocarpus.jpg]

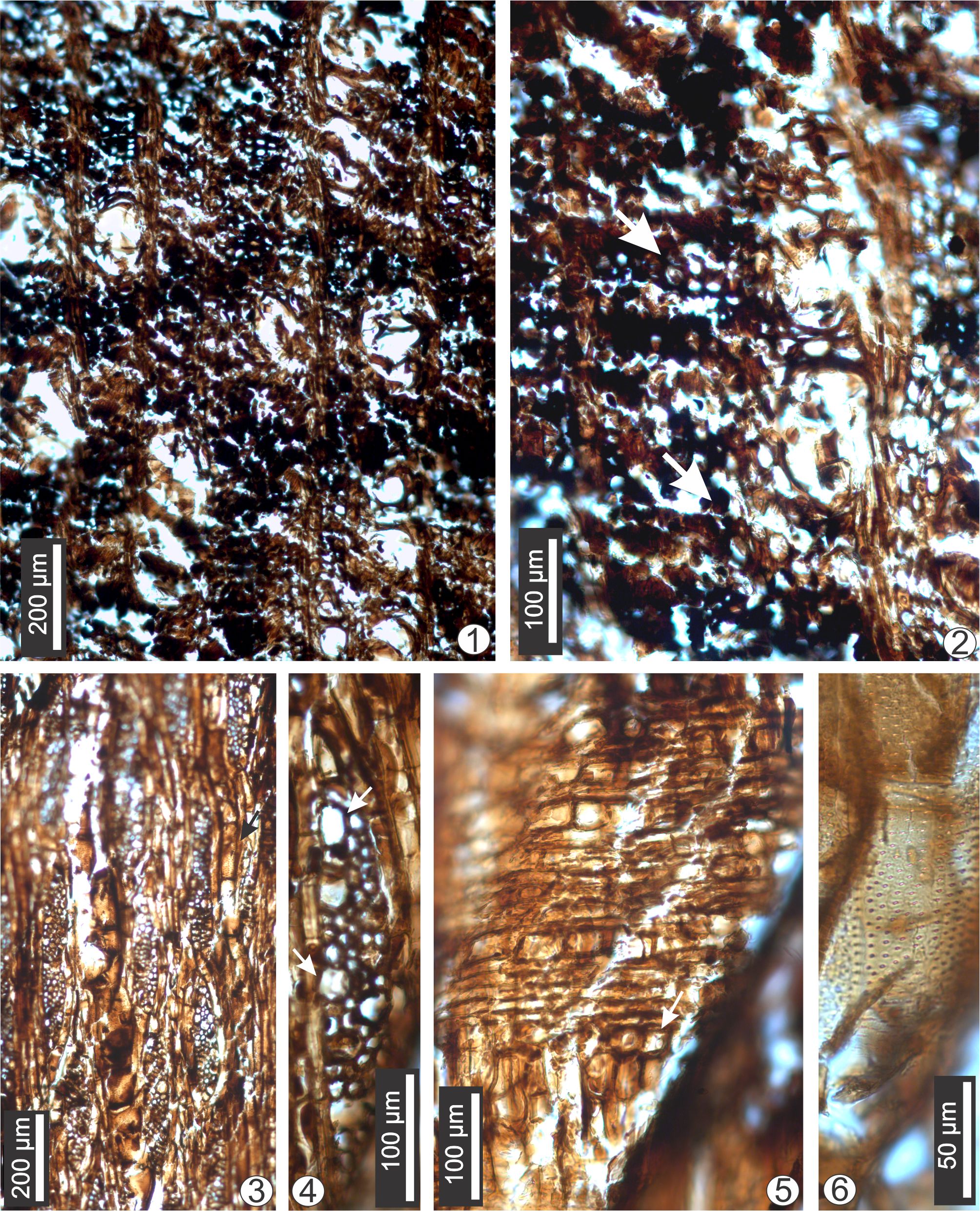

Supplement: File S1 — Supporting Information. Text S1: Systematic description of sub fossil logs. Figure A: Anatomical details of Artocarpus sp. cf. A. lacucha Buch-Ham. Figure B: Anatomical details of Careya arborea Roxb. Figure C: Anatomical details of Diospyros sp. cf. D. bourdilloni Brandis. Figure D: Anatomical details of Dipterocarpus sp. cf. D. indicus Teysm. ex Miq. Figure E: Anatomical details of Neolamarckia sp. cf. N. Cadamba. Figure F: Anatomical details of Rhizophora sp. cf. R. mangle L. (ZIP) [file pone.0093596.s001.zip › File S1 24.3.14/Figure B Careya .jpg]

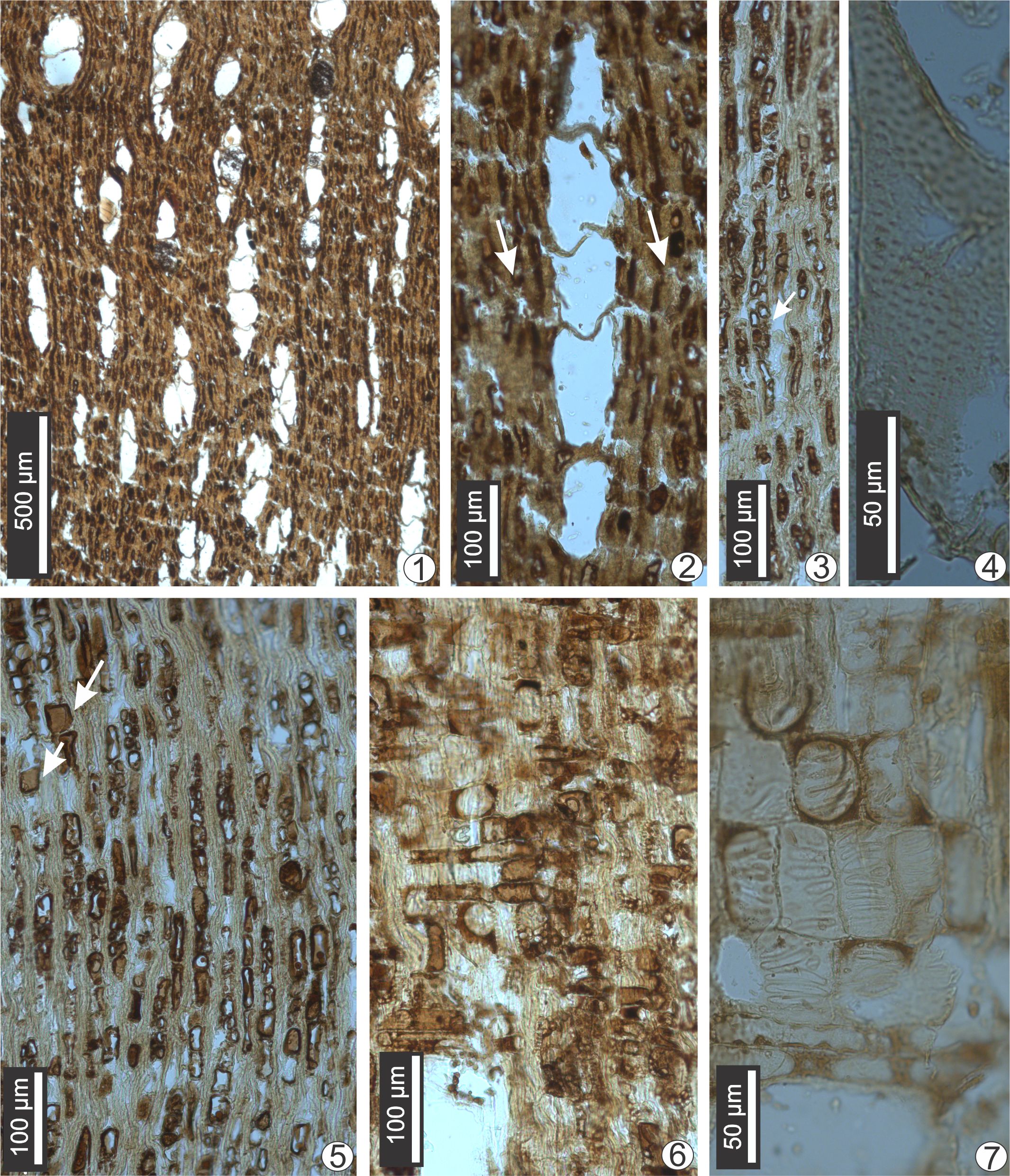

Supplement: File S1 — Supporting Information. Text S1: Systematic description of sub fossil logs. Figure A: Anatomical details of Artocarpus sp. cf. A. lacucha Buch-Ham. Figure B: Anatomical details of Careya arborea Roxb. Figure C: Anatomical details of Diospyros sp. cf. D. bourdilloni Brandis. Figure D: Anatomical details of Dipterocarpus sp. cf. D. indicus Teysm. ex Miq. Figure E: Anatomical details of Neolamarckia sp. cf. N. Cadamba. Figure F: Anatomical details of Rhizophora sp. cf. R. mangle L. (ZIP) [file pone.0093596.s001.zip › File S1 24.3.14/Figure C Diospyros.jpg]

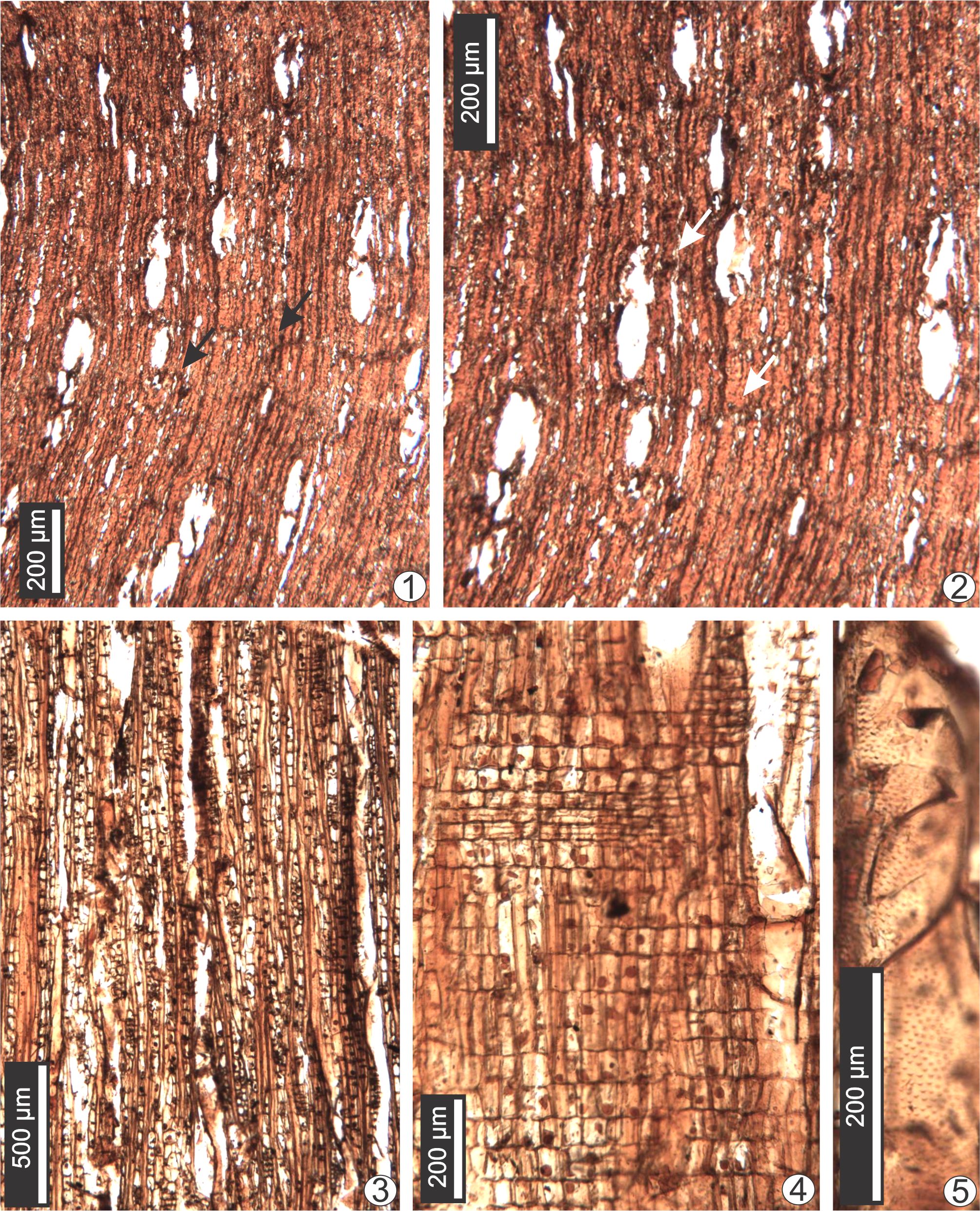

Supplement: File S1 — Supporting Information. Text S1: Systematic description of sub fossil logs. Figure A: Anatomical details of Artocarpus sp. cf. A. lacucha Buch-Ham. Figure B: Anatomical details of Careya arborea Roxb. Figure C: Anatomical details of Diospyros sp. cf. D. bourdilloni Brandis. Figure D: Anatomical details of Dipterocarpus sp. cf. D. indicus Teysm. ex Miq. Figure E: Anatomical details of Neolamarckia sp. cf. N. Cadamba. Figure F: Anatomical details of Rhizophora sp. cf. R. mangle L. (ZIP) [file pone.0093596.s001.zip › File S1 24.3.14/Figure E Neolamarckia.jpg]

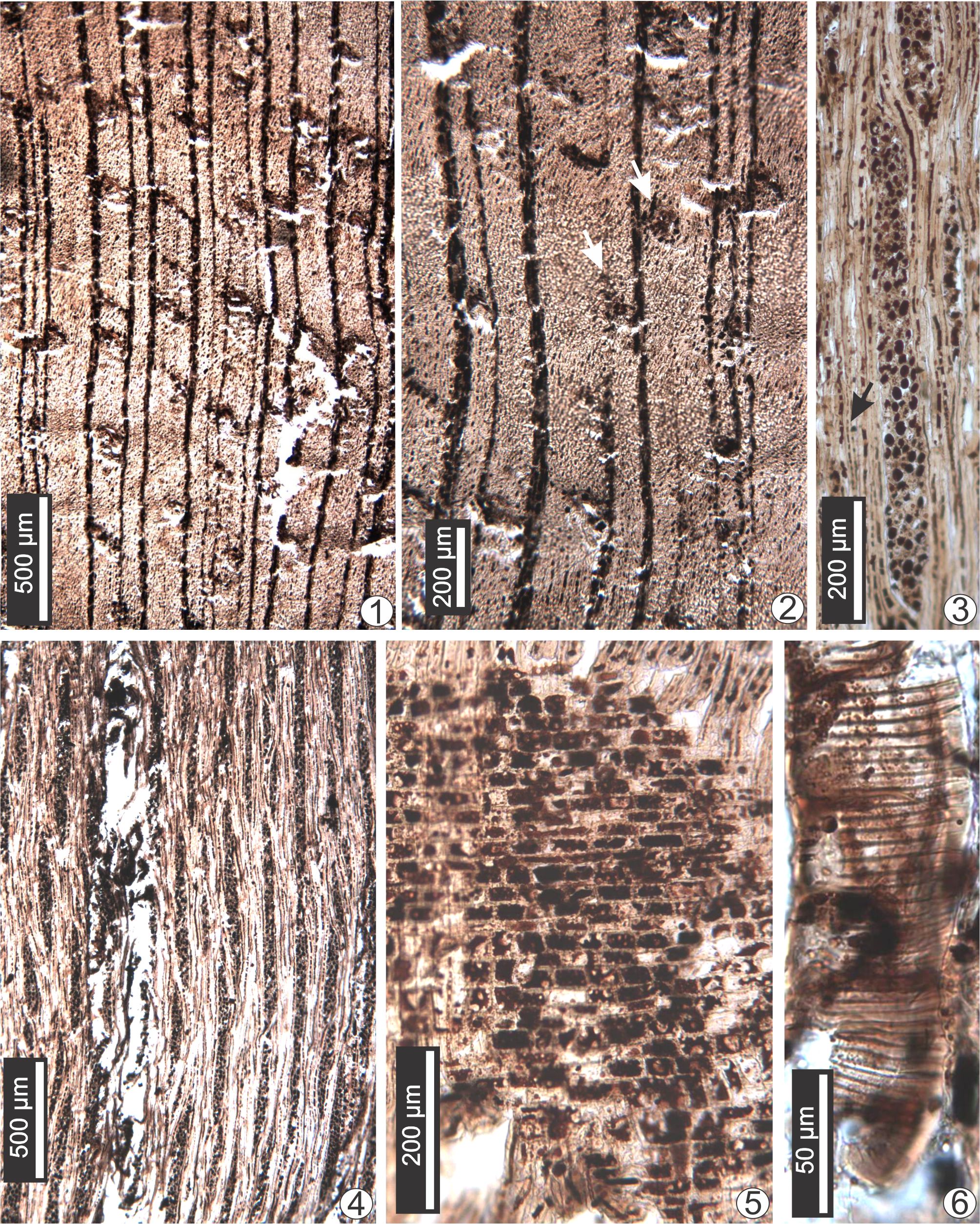

Supplement: File S1 — Supporting Information. Text S1: Systematic description of sub fossil logs. Figure A: Anatomical details of Artocarpus sp. cf. A. lacucha Buch-Ham. Figure B: Anatomical details of Careya arborea Roxb. Figure C: Anatomical details of Diospyros sp. cf. D. bourdilloni Brandis. Figure D: Anatomical details of Dipterocarpus sp. cf. D. indicus Teysm. ex Miq. Figure E: Anatomical details of Neolamarckia sp. cf. N. Cadamba. Figure F: Anatomical details of Rhizophora sp. cf. R. mangle L. (ZIP) [file pone.0093596.s001.zip › File S1 24.3.14/Figure F Rhizophora.jpg]
